# OstemiR: A Novel Panel of MicroRNA Biomarkers in Osteoblastic and Osteocytic Differentiation from Mesencymal Stem Cells

**DOI:** 10.1371/journal.pone.0058796

**Published:** 2013-03-22

**Authors:** Takanori Eguchi, Ken Watanabe, Emilio Satoshi Hara, Mitsuaki Ono, Takuo Kuboki, Stuart K. Calderwood

**Affiliations:** 1 Department of Oral Disease Research, National Center for Geriatrics and Gerontology, Obu, Japan; 2 Division of Molecular and Cellular Biology, Department of Radiation Oncology, Beth Israel Deaconess Medical Center, Harvard Medical School, Boston, Massachusetts, United States of America; 3 Department of Bone and Joint Disease, National Center for Geriatrics and Gerontology, Obu, Japan; 4 Department of Oral Rehabilitation and Regenerative Medicine, Okayama University Graduate School of Medicine, Dentistry and Pharmaceutical Sciences, Okayama, Japan; The University of Adelaide, Australia

## Abstract

MicroRNAs (miRNAs) are small RNA molecules of 21–25 nucleotides that regulate cell behavior through inhibition of translation from mRNA to protein, promotion of mRNA degradation and control of gene transcription. In this study, we investigated the miRNA expression signatures of cell cultures undergoing osteoblastic and osteocytic differentiation from mesenchymal stem cells (MSC) using mouse MSC line KUSA-A1 and human MSCs. Ninety types of miRNA were quantified during osteoblastic/osteocytic differentiation in KUSA-A1 cells utilizing miRNA PCR arrays. Coincidently with mRNA induction of the osteoblastic and osteocytic markers, the expression levels of several dozen miRNAs including miR-30 family, let-7 family, miR-21, miR-16, miR-155, miR-322 and Snord85 were changed during the differentiation process. These miRNAs were predicted to recognize osteogenic differentiation-, stemness-, epinegetics-, and cell cycle-related mRNAs, and were thus designated OstemiR. Among those OstemiR, the miR-30 family was classified into miR-30b/c and miR-30a/d/e groups on the basis of expression patterns during osteogenesis as well as mature miRNA structures. *In silico* prediction and subsequent qRT-PCR in stable miR-30d transfectants clarified that context-dependent targeting of miR-30d on known regulators of bone formation including *osteopontin*/*spp1, lifr, ccn2*/*ctgf*, *ccn1*/*cyr61, runx2, sox9* as well as novel key factors including *lin28a, hnrnpa3, hspa5/grp78, eed and pcgf5*. In addition, knockdown of human OstemiR miR-541 increased *Osteopontin*/*SPP1* expression and calcification in hMSC osteoblastic differentiation, indicating that miR-541 is a negative regulator of osteoblastic differentiation. These observations indicate stage-specific roles of OstemiR especially miR-541 and the miR-30 family on novel targets in osteogenesis.

## Introduction

RNA transcripts from the genome spontaneously form stem, loop and/or bulge structures, and many of these have been reported to act as primary miRNA (pri-miRNA). Drosha, an RNase III in mammalian, then cuts the pri-miRNA to create pre-miRNA that has a simpler stem-bulge structure. Exportin-5 subsequently exports the pre-miRNA to the cytoplasm [1], [2]. Dicer, another RNase III, next cuts the pre-miRNA and creates double strand RNA (dsRNA). Each strand of the dsRNA is a 21–25mer nucleotide, and acts as miRNA by forming the RNA-induced silencing complex (RISC) along with the Argonaute protein [3]. The miRNAs can then hybridize partially with mRNAs, inhibit mRNA translation [4] and promote mRNA degradation [5], [6]. Small RNAs have been also reported to regulate transcription in fission yeast [7], in Drosophila [8] and in human [9]. Moreover, miRNA have also been detected in exosomes suggesting a potential role in regulating gene expression in a paracrine or autocrine manner [10]. Recent studies also imply that miRNA may be involved in a feedback system of gene expression where the miR-371-373 cluster was controlled by Wnt target beta-catenin/TCF and this miRNA cluster could then inhibit DKK1, a Wnt/LRP inhibitor [11].

Osteoblasts are crucial for bone formation during development and metabolism in adult animals by coupling with osteoclasts and thus playing key roles in osteocytic differentiation. Runx2/Cbfa1, Sp7/Osterix and beta-catenin, which are the master transcription factors for osteocytic differentiation, regulate the differentiation of osteoblasts [12], [13]. In addition, the CCN family proteins are known as crucial growth factors for bone formation [14]. The *ccn2*/*ctgf* gene is inducible by TGF-beta/smad and through TRENDIC [15], [16], and is expressed in mesenchymal/fibroblast and vascular endothelial lineages to induce chondro-, and osteo-genesis in target cells [17], [18]. Recent studies have demonstrated that beta-catenin/T-cell factor (Tcf)/lymphoid enhancer factor (Lef) shares a binding site with sox9, a master transcription factor for chondrogenesis, in the *ctgf*/*ccn2* promoter region and provides stage specific control of *ctgf*/*ccn2* expression [19].

Osteocytes, the terminally differentiated cells derived from osteoblasts, are crucial for the mechanotransduction/mechano-stress response, leading to inhibition of osteoclastic bone resorption, mineralization, and mechanical strengthening of bone while preventing osteoporosis and fracture [20], [21], [22]. Osteoblasts are differentiated cells derived from mesenchymal stem cells or bone marrow stromal cells [23], and can further maturate to form osteocytes, which reside individually in bone cavities called *lacunae* [24]. Dmp1, Fgf-23 and sclerostin are known osteocyte markers [20], [25]. Dmp1, which is a secretory protein, was the first osteocyte marker to be isolated [26]. In addition, FGF-23 plays an important physiological function in mineral homeostasis and bone formation in the body by modulating phosphate excretion and vitamin D activation in the kidney [27], [28]. Finally, sclerostin, the Wnt antagonist and the product of the *sost* gene that inhibits bone formation and plays a causal role in sclerosteosis, is transcriptionally activated by *mef2c* and is another osteocyte marker [29], [30]. Induction of bone formation by PTH/PHT1R-induced downregulation of *SOST* expression is mediated through MEF2 [31].

In this study, we have carried out a series of experiments on KUSA-A1, a murine bone-marrow-derived mesenchymal stem cell line with the potential to differentiate into several different cell types, although being highly oriented towards osteocytic differentiation [32]. KUSA-A1 cells produce rich amounts of alkaline phosphatase (ALP), synthesize bglap/osteocalcin that encodes the bone gamma-carboxyglutamic acid-containing (gla) protein and mineralize very efficiently [32]. The KUSA-A1 cells present Sca-1, CD44, Ly-6C and CD140 markers on the surface [33]. More significantly, subcutaneous injection of KUSA-A1 cells in mice generates ectopic bone formation.

Previous studies have shown that miRNAs regulate osteoblastic differentiation. miRNAs control the readout of the mRNAs encoding Runx2 [33], FAK [35] and Connexin43 [36] and thus regulate osteoblastic differentiation. In our study, we have analyzed alterations in the miRNA expression signature associated with osteoblast maturation and/or osteocytic differentiation utilizing a qRT-PCR-based miRNA array. We then investigated the relationship between osteocytic differentiation and the deduced patterns of miRNA expression.

## Materials and Methods

### Cell culture, osteo-induction

Mouse mesenchymal stem and bone marrow stromal cell (mMSC) line KUSA-A1 and calvaria-derived osteoblastic cell line MC3T3-E1 were provided from the RIKEN cell bank. These cell lines were cultured in DMEM supplemented with 10% FBS in humidified air containing 5% CO2 at 37°C. For osteogenic induction, cells were cultured until confluency, and then L-ascorbic acid phosphate, dexamethasone and beta-glycerophosphate (DS pharma) were added every other day for osteogenic differentiation.

Human bone marrow stromal cells (hBMSCs, hMSCs) were purchased from Lonza (Lonza Group, Walkersville, USA) and maintained in basal medium (Invitrogen, Carlsbad, CA, USA) containing 15% fetal bovine serum (FBS, Invitrogen), 1% penicillin and streptomycin (Sigma, St Louis, MO, USA), 1% L-glutamine (Invitrogen) and 180 nM of ascorbic acid 2-phosphate (Wako Pure Chemical Industries, Osaka, Japan). For induction toward osteogenic lineage, hMSCs were cultured until confluency and medium was then substituted to osteogenic-induction medium, which was consisted of basal medium supplemented with β-glycerophosphate (10 mM, Sigma), dexamethasone (10×10^−8^ M, Sigma) and KH_2_PO_4_. (Katayama Chemical Industries, Osaka, Japan). Osteogenic medium was changed twice a week.

### Alizarin red staining

For alizarin red staining, cells were fixed with 4% PFA for 15 min, washed with distilled water, stained with 1% Alizarin red S staining solution for 20 min, and then washed again to remove excess dye as previously described [37].

### miRNA qPCR array

Total RNA including small RNA was prepared by using Qiazol and miRNeasy mini kit (Qiagen) with DNase treatment. cDNA was synthesized with a stem-loop adaptor by using RT_2_ First Strand kit (SABiosciences). miRNA qPCR array was carried out by using miFinder miRNA PCR array (SABiosciences). The data were analyzed by using miScript array data analysis (SABiosciences).

### mRNA qRT-PCR (realtime PCR)

Primer pairs for mRNA were designed by utilizing the NCBI gene bank, primer 3 software and perfect PCR system (TaKaRa), in which each 5′- and 3′- primer are designed to recognize different exons for avoiding genomic DNA amplification if available. cDNA synthesis was carried out using mixture of random primer and oligo dT. The cDNA was diluted 4-fold in TE0.1 (10 mM Tris and 0.1 mM EDTA). A mixture of samples and step dilutions (10-fold and 100-fold) were used as a standard to clarify amplification efficiency. Realtime PCR was carried out as described previously [15]. Expression levels were normalized to that of the internal control gapdh or S29. Relative mRNA levels among different gene products were obtained utilizing Δ Δ Ct calculation. ΔΔCt method when amplification efficiencies were similar among those.

### miRNA qRT-PCR

Forward primers specific for each mature miRNA, which detect both human and mouse miRNAs, were designed based on sequence of mature miRNA obtained from the miRBase [38]. cDNA was synthesized by A-tailing and reverse transcription using universal primer by using qScript microRNA cDNA synthesis kit (QuantaBiosciences), the *All-in-One* miRNA qRT-PCR kit (GeneCopoeia) or Mir-X™ miRNA first-strand synthesis. cDNA was diluted 4–20 fold in TE0.1 and step dilution was prepared as describe above. Realtime PCR for miRNA was carried out: 95°C for 5 min initially, then 30–50 cycles of 95°C for 5 s and 60°C for 40–60 s, and final dissociation analysis. miRNA levels were normalized to that of the internal control snord48 or snord66.

### miRNA target prediction

TargetScan 5.1 was utilized either for *in silico* search for miRNAs which recognize 3′-untranslated region (3′-UTR) in a specific mRNA of interest or for prediction of target mRNA of specific miRNA of interest [39]. Target of miR-30 family, miR-34 family, let-7 family, miR-15/16 family (including miR-322/424), miR-21 family, miR-541/654 was predicted and selected using cut off score −0.2. Development, RNA regulation, epigenetics, transcription, protein modification-related factors were preferentially selected.

### Statistics

Data was expressed as means±standard deviations, and the statistical significance of differences in mean values was assessed by Student's unpaired *t* tests for KUSA-A1 cells or by one-way ANOVA with Tukey post-hoc correction tests for hBMSC/MSC. Differences among the mean values were considered significant at a *P* of <0.05. Realtime PCR was repeated 4 times for each samples in experiments using KUSA-A1 cells. Total RNA was isolated from independent triplicate culture of hBMSC/MSC and realtime PCR was repeated in triplicate.

### Stable miRNA expression

miRNA sequences of step loop part of pre-miR-21, pre-miR-30d, and pre-miR-322 were obtained from miRBase. The genomic counterparts of the miRNA stem loop were searched using Blast. Hundred base native flank sequences to both upstream and downstream of the miRNA stem loop were added for PCR amplification using following primers: miR21-F, cagctttctttcctagaattggcattaag; miR21-R, ccaaggtataagggctccaagtctcac; miR30d-F, ttattgtttgtcttttccccccaagatg; miR30d-R, ttagaagctgccagcagaagcaagcag; miR322-F, tcctccccactatccaccacaccctg; miR322-R, caggcccttggactgtgtagagtgac. The amplified fragments were cloned into pSMPUW-miR-GFP/Puro (Cell Biolabs, San Diego, CA) and sequenced to verify the contents. Recombinant lentivirus was generated by Lenti-X Lentiviral expression system (Takara Bio, Shiga, Japan) and infected to KUSA/A1 cells for 36 hours, and then the medium was replaced to normal medium. The infected cells were selected by puromycin (2 ug/mL) in 10 days for cloning of miR-21 and miR-30d stable transfectant and in 2 weeks for miR-322 stable cells.

### Anti-miRNA-mediated knock down

Cells were plated in 6 well plates at a density of 2×105 cells with culture medium without antibiotics. On the following day, cells were transfected with anti-miR541miR-541 (20 nM, miRvana^TM^ miRNA inhibitor, Invitrogen) or negative control (20 nM, mirVana™ miRNA Inhibitor negative control #1, Invitrogen) using RNAiMax (Invitrogen). Cells were cultured for 2 days before total RNA extraction, or culture until confluency before osteogenic induction.

## Results

### Osteoblastic and osteocytic differentiation of KUSA-A1 cells

**Figure 1:**
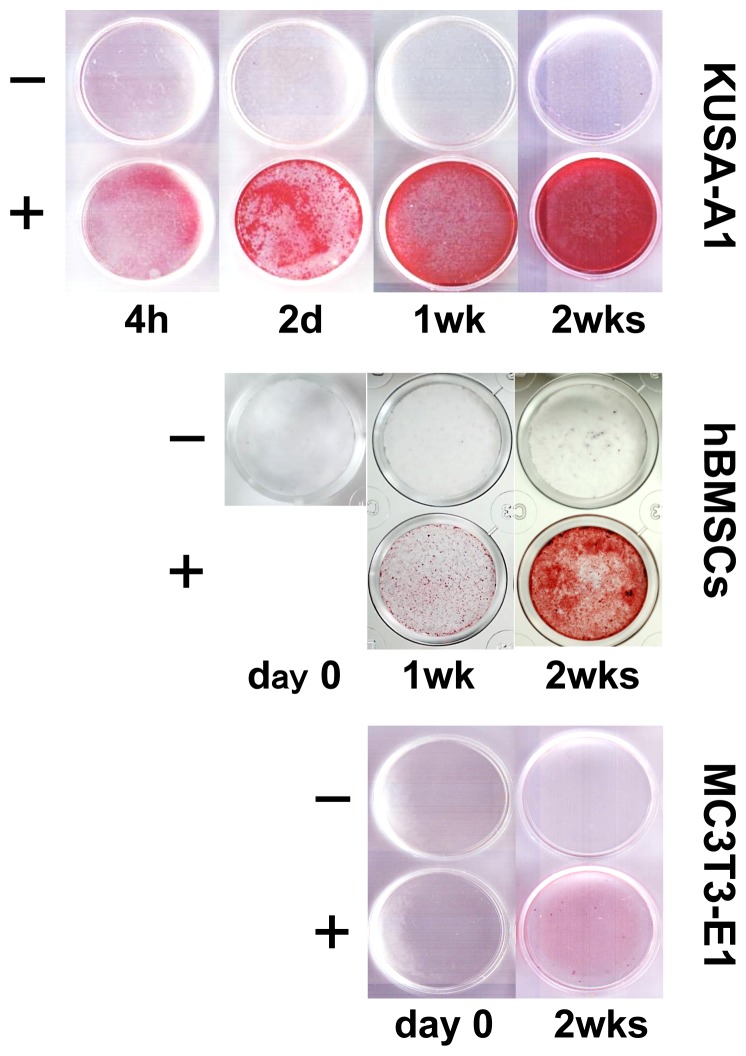
Calcium deposition on KUSA-A1, hBMSC/hMSC and MC3T3-E1 cells during osteogenic differentiation. Cells were cultured with (+) or without (−) L-ascorbic acid phosphate, dexamethasone and beta-glycerophosphate. Calcium deposition was visualized by alizarin red S staining.

**Figure 2:**
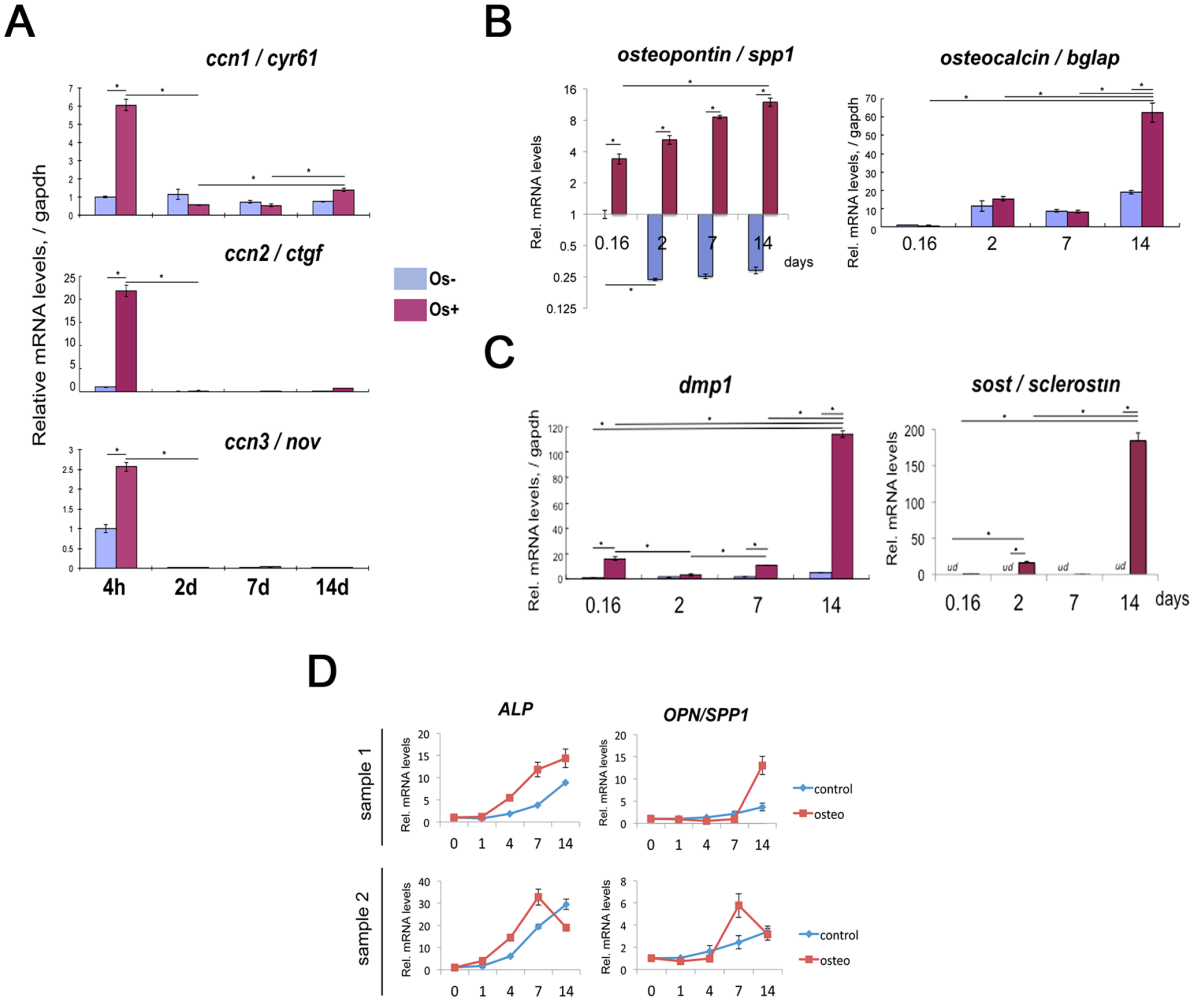
Marker expression pattern during KUSA-A1 mMSC line with (Os+) or without (Os−) induction of osteoblast differentiation. (A) *Cyr61*/*ccn1*, *ctgf*/*ccn2* and *nov*/*ccn3* mRNAs were quantified as osteo-chondro-mesenchymal cell markers in initial differentiation. (B) *Spp1*/*osteopontin* and *bglap*/*osteocalcin* mRNAs were quantified as osteoblastic markers. (C) *Dmp1* and *sost*/*sclerostin* mRNA were quantified as osteocyte markers. *, *P*<0.05 (n = 4). *ud*, undetected. (D) *ALP* and *OPN*/*SPP1* mRNA expression pattern during hBMSC/MSC osteogenesis.

We first investigated KUSA-A1, MC3T3 cells and hBMSC/hMSC in tissue culture as osteoblastic/osteocytic differentiation models (Fig. 1). After adding osteogenic differentiation supplements to cells, calcium was gradually deposited in KUSA-A1 and hMSC culture until 2 weeks after the addition (Fig. 1). The levels of calcium deposition were markedly increased in KUSA-A1 cells after 2 weeks induction, while only mild levels were observed in MC3T3 cells. KUSA-A1 cells were therefore used in the subsequent studies on miRNA signature involved in osteocytic differentiation from MSCs. In order to confirm the differentiation stages, relative expression of markers for mesenchymal, osteoblastic and osteocytic cells were quantified after osteogenic induction. Mesenchymal cell markers *ctgf/ccn2*, *cyr61*/*ccn1* and *nov*/*ccn3* were induced at initial stimulation (4 hrs) but declined as cells differentiated along the osteocyte lineage (4w+: Fig. 2A). The Osteoblast marker *spp1*/*osteopontin*, increased on 4 hours (0.16 day), 2 days, 7 days and 14 days after starting osteogenic induction (Fig 2B), and the *spp1* level remained at a decreased level in confluent cells without osteoinduction. Another osteoblast marker *Bglap*/*osteocalcin* was induced at 2 weeks after osteoinduction compared with non-induced control (Fig 2B). *Dmp1*, an osteocyte marker, was powerfully induced at 4 hours, 7 days and 14 days after osteo-induction (Fig. 2C). Late stage osteocyte marker sost/sclerostin was increased on day 2 and especially on day 14, while under detection limit on other conditions and time points (Fig 2C),

These results suggest that this MSC differentiation model of KUSA-A1 is useful for analysis of either osteoblastic and/or osteocytic differentiation from MSCs.

### Osteoblastic differentiation of hMSC/hBMSC

In order to firstly establish osteoblastic differentiation from hMSC, osteo-induction, calcium staining and quantification of mRNA levels of marker genes were carried out. Calcium was accumulated on hMSC upon osteo-induction around 1 and 2 weeks after the induction gradually as shown by alizaline red S staining (Fig 1), while non-osteo-induced cells were not stained. *ALP* and *SPP1*/*Osteopontin (OPN)* mRNA were quantified in realtime qRT-PCR during osteo-induction. Both *ALP* and *OPN*/*SPP1* were upregulated in two independent experiments with different peak timings. In the first sample, both *ALP* and *OPN/SPP1* peaked on day14, while in the 2nd sample, both *ALP* and *OPN*/*SPP1* peaked on day 7 and decreased thereafter (Fig 2D), indicating that differentiation is slower in sample #1, while faster in sample #2 relatively each other. These data suggest that hMSC/hBMSC differentiated into osteoblasts after the induction.

### Overview of the miRNA array approach

**Figure 3:**
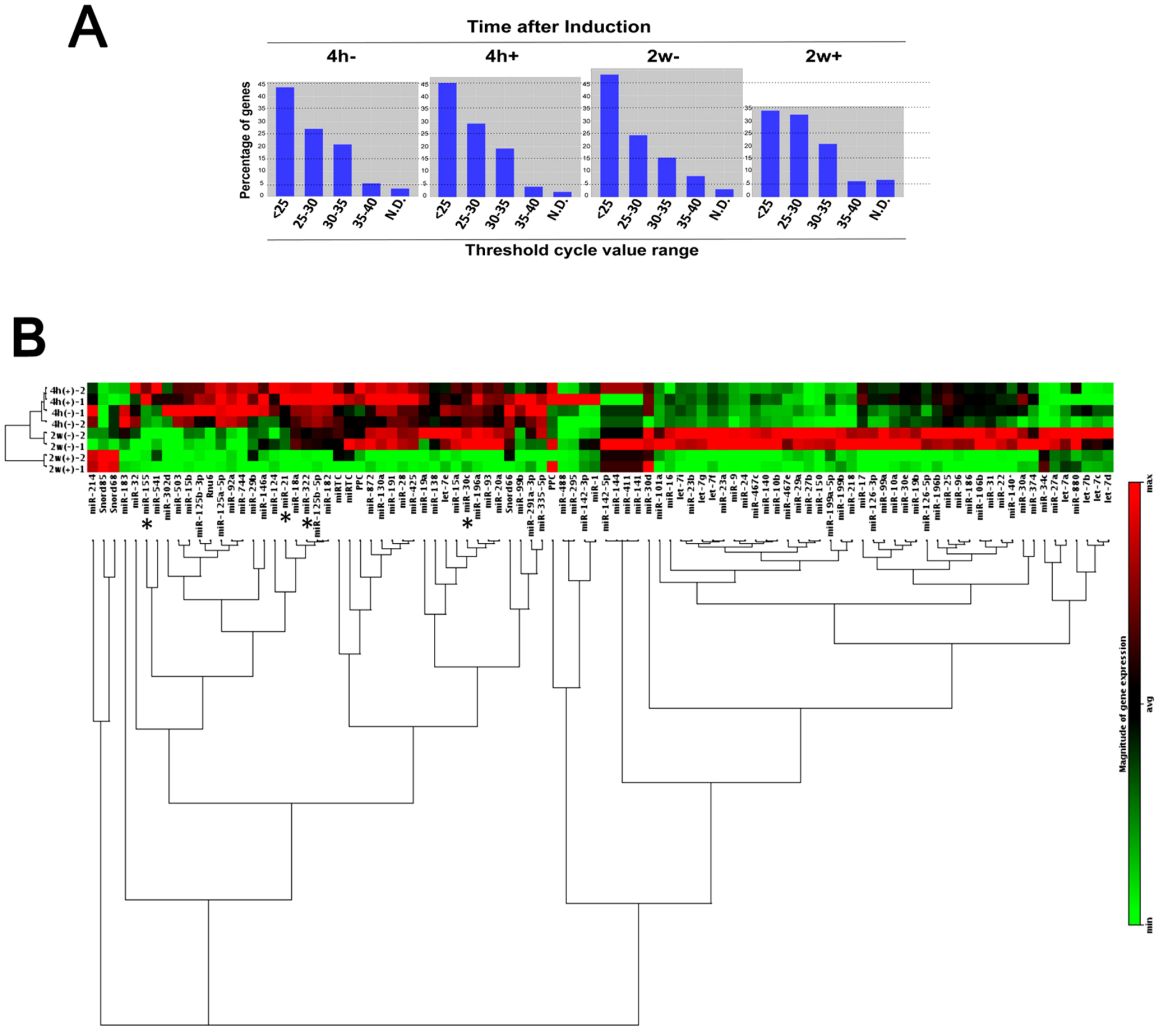
miRNA expression signature during MSC osteocytic differentiation. (A) Difference in miRNA expressions among experimental conditions with or without induction for 4 hours or 2 weeks. (B) Clustergram of miRNA expression in KUSA-A1 cells with or without osteo-induction for 4 hours or 2 weeks. Red and green indicate relatively high and low expression levels, respectively.

To investigate novel miRNAs that could potentially regulate osteoblastic/osteocytic differentiation and stemness (OstemiR), a screening with a qRT-PCR-based miRNA array was utilized. We first obtained an overview of changes in overall miRNA levels. Total miRNA expression levels after osteo-induction for 2 weeks were significantly lower than observed in other conditions (Fig. 3A). All the tested miRNAs were then analyzed by clustergram and the findings indicated that most miRNAs were expressed at a reduced level in mature osteocytes (Fig. 3B). Based on the results of the clustergram, miRNAs were then categorized into eight groups (Table 1). The most abundant group was the miRNAs that increased in level after prolonged culture without osteo-induction (2w−). miRNA downregulated by two weeks osteo-induction included members of the let-7 and miR-30 families (miR-30a/d/e) (Table 1). Thus, the total amount of miRNA is reduced in osteocytes compared to osteoblast precursors, and release of translational repression may be involved in induction of osteocyte-specific factors and the osteocytic phenotype.

**Table 1:** miRNA groups categorized by expression pattern.

Expression	miRNA	Possibility #1	Possibility #2
high in 4h, low in 2w	miR-15b, 125a-5p, 92a, 744, 29b, 146a, 124, 21, 18a, 322, 125b-5p, 182, Rnu6	Stemness marker	Differentiation inhibitor
high in 2w, low in 4h	30d	Differentiation marker	Stemness inhibitor
high in 4h+, low in 4h−	miR-155, miR-541, miR-21	Initiator of Differentiation	Inhibitor or maintiner of stemness
high in 4h−, low in 4h+	miR-30d	Inhibitor of Initiation	Inhibitor or maintainer of stemness
high in 2w+, low in 2w−	No miRNA in this pattern was observed.	Osteocyte marker	Stemness inhibitor
high in 2w−, low in 2w+	miR-18a, 322, 125b-5p, 182, 872, 130a, 191, 28, 425, 196a, 93	Osteocyte negative marker	Stemness marker
high only in 2w+	Snord85	Osteocyte marker	Stemness inhibitor
high only in 2w−	miR-101a, 16, 23b, 23a, 9, 24, 467c, 140, 10b, 467e, 29a, 27b, 150, 199a-5p, 199b, 218, 17, 126-3p, 99a, 10a, 30e, 19b, 126-5p, 196b, 25, 96, 186, 106b, 31, 22, 140, 30a, 374, 34c, 27a, 880. let-7i, 7g, 7f, 7a, 7b, 7c, 7d	Osteocyte negative marker	Stemness marker

Possible functions of miRNAs were shown in right.

**Figure 4:**
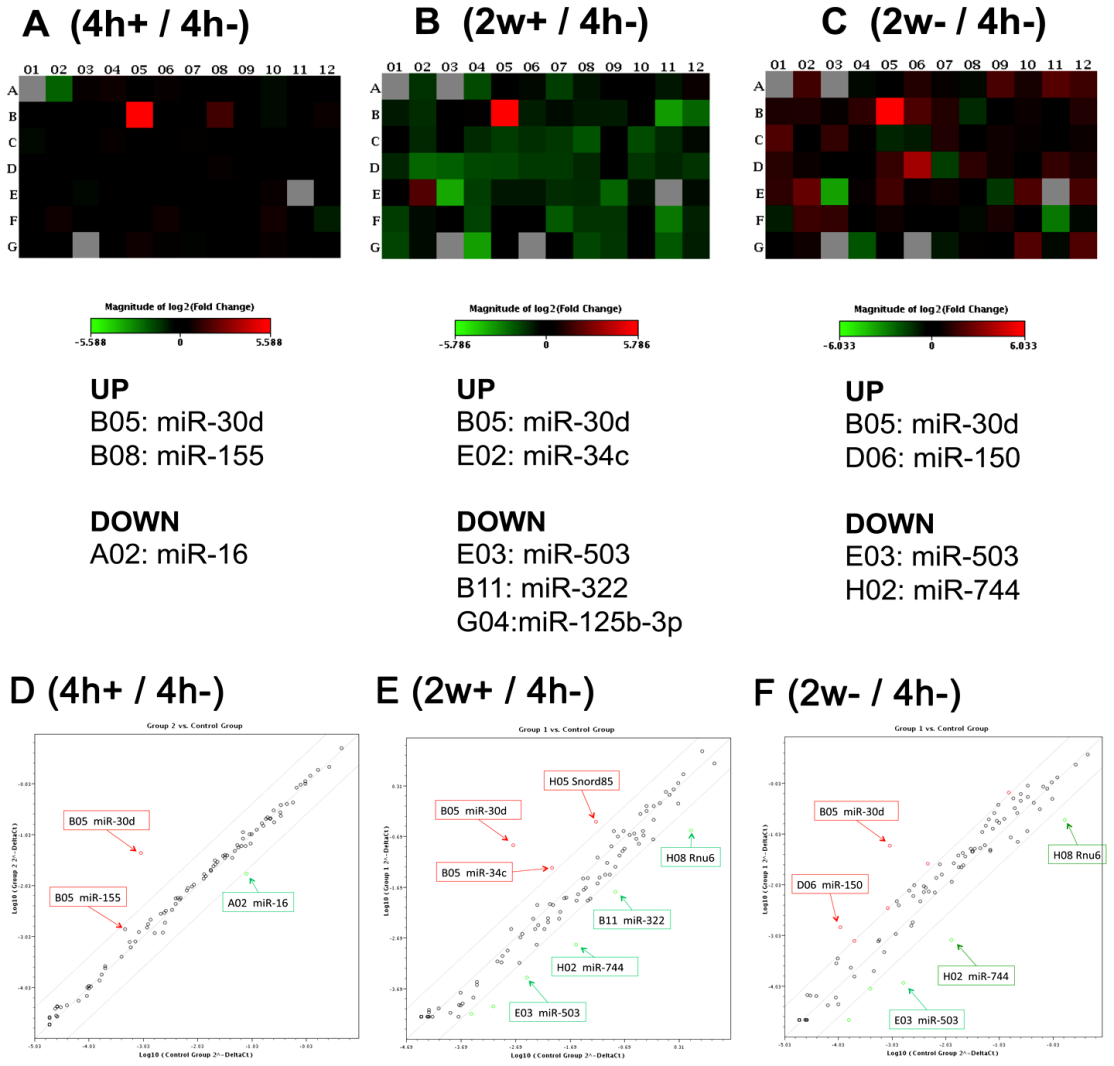
Heat maps and scatter plot analysis of miRNA array. Upregulated miRNA (red) and downregulated miRNA (green) were colored and listed. miRNA expression after 4 hours of osteo-induction (4h+, A, D), osteo-induction for 2 weeks (2w+, B, E) or 2 weeks of long culture (2w−, C, F) were compared to non-induction control (4h−).

For a better understanding of OstemiR, the miRNAs involved in osteocyte differentiation, heat maps were created to compare miRNA expression levels between experimental conditions of induction (4h and 2w+/− differentiation stimulus). The expression level of miR-30d was higher in the 4h+, 2w- and 2w+ condition than in the 4h− condition (Fig. 4A–F). Indeed, by a single stimulation for osteocytic differentiation, not only the miR-30d but also miR-155 was induced (Fig. 4A, D). The expression level of miR-16 was decreased by the single stimulation (Fig. 4A, D). After repeated osteo-induction (2w+), miR-30d and miR-30c were induced, and the expression levels of miR-503, miR-322 and miR-125b-3p were the most powerfully repressed (Fig. 4B, E). miR-30d and miR150 as well as other miRNAs were induced by long-term culture for 2 weeks in the absence of differentiation stimulus, while miR-503 and miR-744 were reduced by the long-term culture (Fig. 4C, F).

### Standardized quantification of miRNA expression

**Figure 5:**
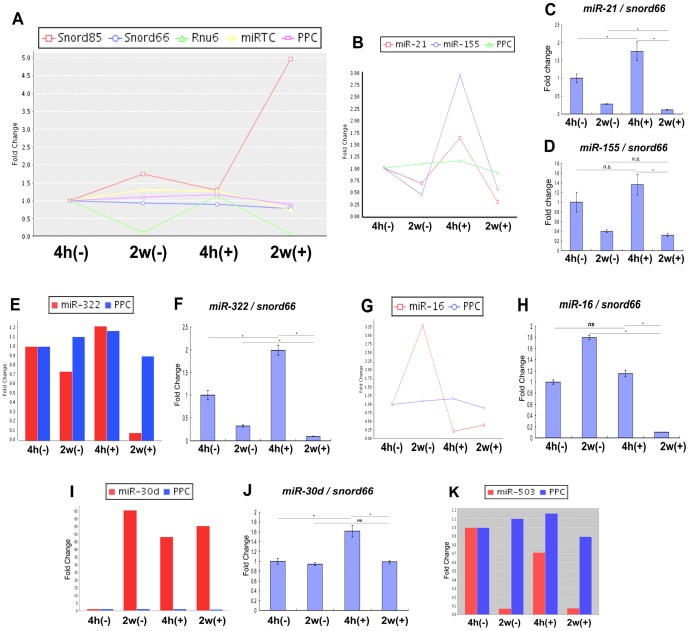
Quantification of miRNA expression levels during osteocytic differentiation. (A) Expression of internal controls for miRNA quantification. Snord85, Snord66 and Rnu6 levels during osteogenesis of KUSA-A1 were quantified in PCR array. miRTC, miRNA reverse transcription control. PPC, positive PCR control. (B–K) Quantification of miRNA expression levels by using miRNA array (B, E, G, I, K) or qRT-PCR (C, D, F, H, J). Values were normalized to snord66 levels (C, D, F, H, J). *, *P*<0.05 (n = 4). *n.s.*, not significant.

In order to establish an internal control for miRNA quantification in the KUSA-A1 cell during the conditions of osteocytic differentiation, Snord85, Snord66, Rnu6, miRTC and PPC expression patterns were examined from the results of the miRNA array (Fig. 5A). Among these candidate control RNAs, the expression patterns of Snord66 and PPC were found to be the most stable in all-experimental conditions, while Rnu6 was not stably expressed, and Snord85 levels became elevated after 2 weeks of osteo-induction. Therefore, Snord66 or PPC were adopted as internal controls for miRNA quantification in the KUSA-A1 osteocytic differentiation system in later experiments. In addition, Snord85 was established as a novel marker of osteocytic differentiation.

We next attempted to confirm the reliability of the results of the miRNA array analysis, designed specific primers for each miRNA, and quantified miRNA levels using qRT-PCR. Expression of both miR-21 and miR-155 was stimulated by the initial differentiation stimulus (4h+) although expression was suppressed at the terminal differentiation stage (2w(+) as indicated by both qRT-PCR and miRNA array (Fig. 5B, C, D). miR-322 was investigated by the same methodology, and shown to be decreased at 2 weeks after osteo-induction in both the qRT-PCR and miRNA array (Fig. 5E, F). miR-16 was also repressed by osteogenic stimulus as quantified by either miRNA array or qRT-PCR (Fig. 5G, H). miRNA array analysis showed that miR-30d was induced by single stimulation (4h+), repeated stimulation (2w+) and prolonged culture in the absence of stimulation (2w−). However, miR-30d was increased only by single stimulation as indicated by qRT-PCR (Fig. 5I, J). Overall, these results indicate that while the qRT-PCR-based miRNA array is useful and reliable for screening, individual qRT-PCR is more reliable and flexible for the assay of single-peaked amplification of cDNA in detailed kinetic analysis.

### Prediction of targets for, and functions of OstemiR during osteoblastic/osteocytic differentiation

The functions and targets for OstemiRs, whose expression levels were significantly changed during osteocytic differentiation, were predicted by analysis of database (Table 2). These OstemiRs were predicted to recognize mRNAs encoding a number of key transcription factors for osteocytic differentiation. These included: (*runx2, sox9, sox5, smad* family, *msx1, tcf, jun* and *fos*), transcription factors for pluripotency (*sox2, klf5*/*klf, mycn*), EMT-related factors (*snail, zeb2, claudin-1*/*2*), essential growth factors for osteocytic differentiation and their receptors (*wnt/lrp, fgf/fgfr, bmp/gdf/bmpr/tgfbr/actvr, ihh, lifr, notch, igf/igfr, insr*), matrix-related molecules (*reck, timp*), neuronal markers (*gfap*), epigenetic factors (*mll, jmj, jhdm, hdac/sirt1, tet, jarid, mbd, ncoa1*), cell cycle proteins (*cyclin/cdk*) and heat shock proteins (*hsp, dnaj*) (Table 2). The cohort of miRNA, which was upregulated during osteoblast maturation, including miR-30d, miR-155, miR-21 and miR-16, constitutes a marker of osteocytic differentiation and these miRNA may possibly repress stemness maintenance in osteoblasts. Both miR-34c and miR-16, which increased at 2w+, the stage of osteocytes, are possibly osteocyte markers and repressors of osteoblast-maintaining genes.

**Table 2:** OstemiR expression pattern and predicted targets.

miRNA	Results in miRNA array	Results in individual qRT-PCR	Possible actions	Possible target signals	Development and stemness-related	Epigenetic and protein modification-related	Cell-cycle-related	Others
miR-30d	Low in 4h−	High in 2h+	Repressing stemness or osteoblast factors	Wnt, FGF, BMP/TGFb, Runx2, Osx	RUNX2, SOX9, LRP6, SMAD2, SMAD1, NOTCH1, NOV, IGF, IGF2R, IGF1R,BDNF,	MLL, JMJD1A, SIRT1, HDAC5, ITGA5, NCOA1, HOXB8, FOXO3, SENP5, MBD6, TET1,TET3	CCNE2, P15RS, CCNT2, CCNK, Cdk6, CCNJ-like	ITGA4,SNAI1, ITGB3,KLF9, KLF11, ZEB2, HSPA5,
miR-155	High in 4h+, Low in 4h−	Similar with array data	Repressing stemness or osteoblast factor	Wnt, FGF, BMP/TGFb, Runx2, Osx	LRP1B, TCF4, SP1, SMAD2,GDF6, FGF7, FOS, SMAD1, SP3, TGFBR2, ACVR2B,	JHDM1D, TP53INP1, JARID1B, SIRT1,	DMTF (cyclin D binding myb-like transcription factor 1), CCND1	SMARCAD1, KLF3, Claudin-1, DNAJB1,
miR-21	High in 4h+, Low in 4h−	Similar with array data	Repressing stemness or osteoblast factor	Wnt, FGF, BMP/TGFb, Runx2, Osx	BMPR2, ACVR1C, SKI, LIFR, TGFBI, SOX5, FGF1, SMAD7, LRP6, KLF5, SOX2, TGFBR2, MSX1, TET1,	JHDM1D,	Cdk6	RECK, ITGB8, KLF3, TIMP3, KLF12,
miR-34c	High in 2w+, low in 4h−		Repressing stemness or osteoblast factor	Wnt, FGF, BMP/TGFb, Runx2, Osx	MYCN, FGF23, NOTCH1/2, ACVR2B, SMAD4, FOSB	JHDM1D,	CCNE2, Cdk6	ITGB8, ITGA10,CTNND2, BCL2, TGIF2, PDGFRA, HSPA1B,
miR-16*	High in 4h−, Low in 4h+/2w+	Low in 2w+	Repressing stemness or osteoblast factors	Wnt, FGF, BMP/TGFb, Runx2, Osx, CCN	INSR, ACVR2B, SMURF1, FGF7, LRP2/6, FGFR1, SMAD7, WNT3A, NOTCH2, SMAD5, SMAD3, IHH, WNT4, TGFBR3, LRP1B, TCF3, SMURF2, BMPR1A, IGF2R, WNT7A, BMP8A, WISP1, SOX5, WNT5B, ACVR2A, BDNF, HSPG2, IGF1R, IGF1, GHR, PTH,	SIRT4, SUMO3, ITGA10,LITAF, FOXO1, TET3,	CCNE1, DMTF, CCND2, CCNT2, Runx1T1 (CCND-related), CDK5R1, CCNM2, CCND1, CCND3, CCNJL, Cdk6,	MYB,GFAP,MYBL1, VEGFA, RECK, CREBZF, CTNNBIP1, FOSL1,RICTOR, CLDN2, DNAJB4,
miR-322/424*	High in 4h−, Low in 2w+	High in 4h+, Low in 2w+						
miR-503	High in 4h−, Low in 2w+/−		Repressing stemness or osteoblast factors	BMP/TGFb, Runx2, Osx, CCN	SMURF1, FGF7, INSR, ACVR2B, BMPR1A, SMAD7, SMURF2, WNT3A, WNT4, SOX5, IHH, HSPG2, IGF1, IGF1R,	JARID2,	CCND2, CCNE1, CCND1, Cdk5R1, CCNM2, CCND3,	RECK, VEGFA, MYBL1, FOSL1,
miR-541					ADAMTS7, WNT11, TIMP2, ELN, SP1	EIF2C1/AGO1, WDR82, USP9X, JMJD3		BAZ1B, ITGA3
miR-744	High in 2w+/−, Low in 4h−		Repressing stemness factors		JUNB, TGFB1			LRP3

From the expression patterns of miRNA, actions and target mRNA were expected. Target genes in Homo sapiens were predicted by using TargetScan 5.2. Bone-, stemness-, epigenetics and cell-cycle-related target mRNAs were selected and shown. *miR-16 and miR-322/424 share targets.

### Prediction of miRNAs that target osteo-regulators

By the prediction of miRNAs that target osteo-regulators, miRNA recognition sites in the 3′-UTR regions in the target mRNAs were predicted and classified into groups that were: (a) conserved among all vertebrates, (b) conserved only among mammalian species or (c) poorly conserved. The conserved miRNA recognition sites among all vertebrates (a) and only among mammalians (b) are described below. A let-7/miR-98 recognition site was predicted in the 3′-UTR region of *dmp1* mRNA with broad conservation among vertebrates (Fig. S1). Since all members of the let-7 family are downregulated during osteocytic differentiation (Table 1) it was predicted that the downregulation of the let-7 family could be associated with an eventual repression of the *dmp1* gene. In addition, miR-30d was induced by osteo-induction (Fig. 5J), and miR-30 family recognition sites were found in the 3′-UTR regions of the *runx2* and *nov*/*ccn3* mRNAs (Fig. S2, S3). Moreover, the miR-30 family was predicted to recognize *sox9, lrp6, smad2, smad1, notch1, bdnf* and a number of epigenetic factors (Table 2). These findings suggest that members of the miR-30 family could play an essential role in osteocytic differentiation. Moreover, among miRNAs that were predicted to recognize the 3′-UTR of *ctgf*/*ccn2*, an osteo−, chondrogenesis factor, and miR-18ab and miR-19 are also OstemiR, which increased during osteocytic differentiation (Fig. S4, Table 1).

### miR-30 expression pattern during KUSA-A1 MSC osteocytogenesis

**Figure 6:**
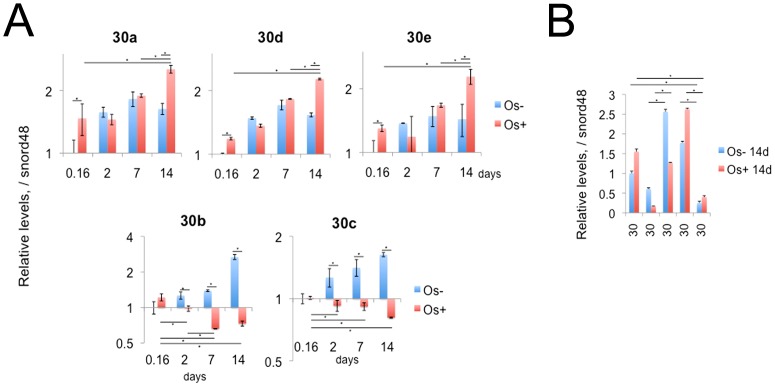
Mature miR-30 quantification during osteocytogenesis. (A) miR-30 family expression pattern in KUSA-A1 mMSC line with (red bars, Os+) or without (blue bars, Os−) osteoinduction. Values were normalized to snord48 levels. (B) Relative expression levels among miR-30 family members on day 14. Note the different expression levels: miR-30d>30a>30e: miR-30c>30b. *, *P*<0.05 (n = 4).

In the miRNA PCR array, miR-30d showed an increased expression level in osteocytogenesis of KUSA-A1. Since miR-30 family members are homologous (Fig 6A) and possibly share targets, we further investigated the miR-30 family expression patterns at four time points with or without osteo-induction. As a result, miR-30a, miR-30c and miR-30d were highly expressed compared with miR-30b or miR-30e (Fig. 6B). All the miR-30 members once reduced during osteoblastic differentiation stage on day 2 and day 7. Among those members, miR-30a/d/e were increased on day 14 around a late osteocytic stage (Fig 6A). These data suggest that miR-30 members could be repressing targets at the MSC and osteocytic stages, while repression on target mRNA may be relieved during the intermediate osteoblastic stage.

### Prediction of miR-30 targeting

**Figure 7:**
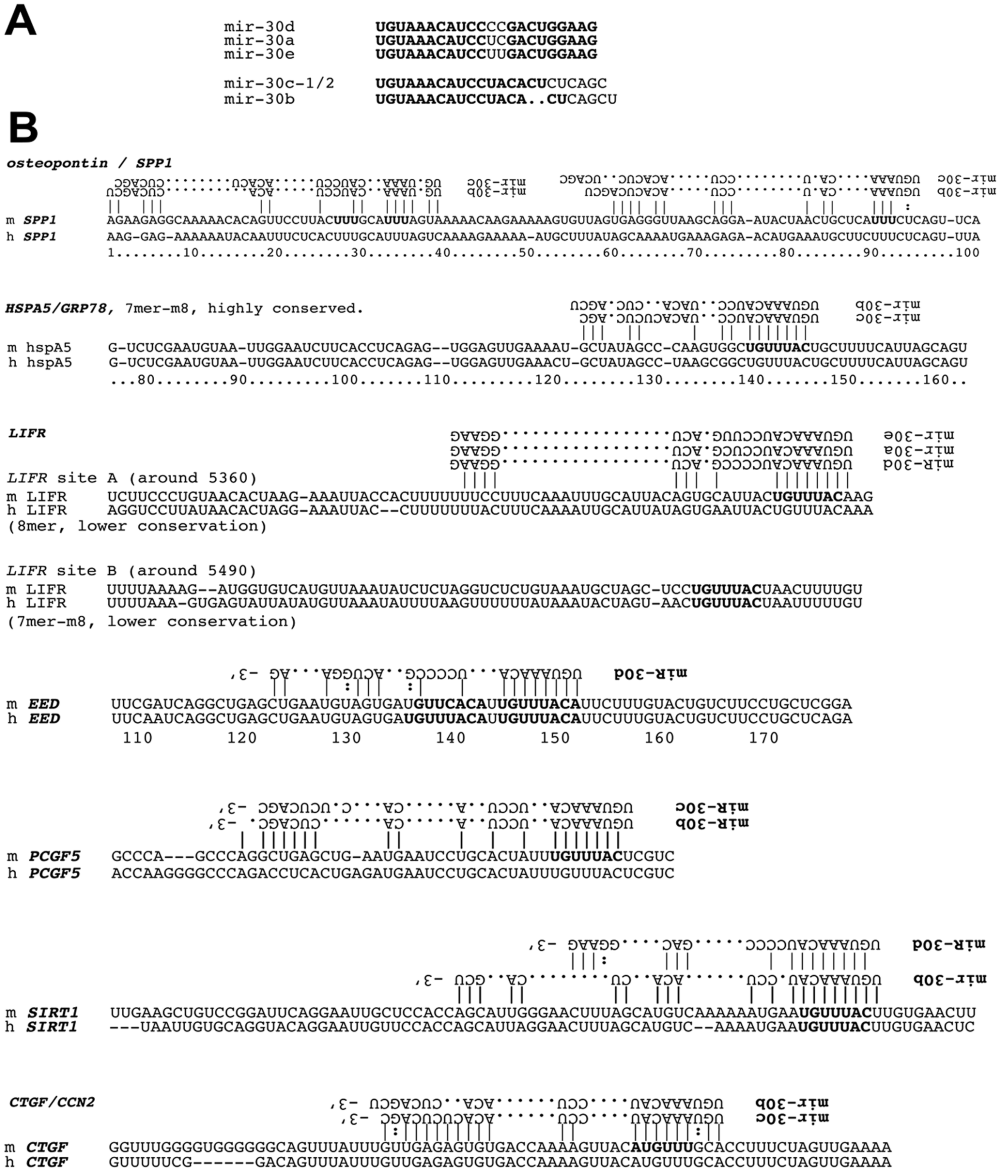
miR-30 targeting prediction. (A) List of mature miR-30 family members. Homologous nucleotides among miR-30a/d/e or between miR-30b/c were shown in bold. (B) Base pairing prediction between miR-30 and target sequences in the 3′-UTR of *SPP1/osteopontin, HSPA5/GRP78, LIFR, EED, PCGF5, SIRT1 and CTGF/CCN2.* Note that targeting can be shared among some family members. Positions in 3′-UTR were shown below the sequences.

miR-30 targets were predicted using TargetScan. Putative targets with strong scores were selected by a cut-off score −0.4. Factors related to development (*runx2, eed, sox9, lifr, lrp6, ctgf*), epigenetics (*wdr82, brwd1, pcgf5, eed*), transcription and cell cycle (*zbtb41, zbtb44, ccnt2, ccne2*), RNA biogenesis (*hnRNP3, Lin28A, Lin28B, helz*), post-translational protein modification (*snx16, yod1*) and known targets (*runx2, ctgf, grp78/hspa5*) are shown in the list (Table 3). These predictions appear to be specific to each of the miR-30 members; however, 11 nt of the 5′ seed sequence in miR-30 family members are common and the mature miR-30s sequences are quite homologous among miR-30a/d/e or between miR-30b/c (Fig 7A), indicating shared and distinctive targets among miR-30 members.

**Table 3:** List of predicted miR-30 targets.

miRNA	targets	score	Note	Class	Protein locality
miR-30b	*SNX16*	−1.2	Phosphatidil inositol binding (Phox) domain, protein trafficing.	Phos Sig	C
miR-30b	*Runx2*	−1	Master transcription factor for osteoblast differentiation. Known target of miR-30.	Dev, Txn	N
miR-30b	*hnRNPA3*	−0.96	hnRNPA family directly bind to mRNA for nuclear export. hnRNPA1 binds Pri-let-7a-1and inhibit Drosha cleavage.	RNA	N/C
miR-30b	*EED*	−0.9	Embryonic ectoderm development. polycomb group (PcG), Component of PRC2/EED-EZH2 complex. K9/K27 methylation for repression. Control ES cell self-renewal loop with Sox2. interact with integrin beta7 (may mediate integrin signal), interact with HDAC for histone deacetylation. WD protein associated, miR-30-specificity.	Dev, Epige, Stem	N, Chro
miR-30b	*CCNE2*	−0.84	G1/S transition	Cell cycle	N
miR-30b	*YOD1*	−0.7	DeUbiquitination enzyme	Protein Modi	
miR-30b	*WDR82*	−0.61	WD repeat domain protein. let-7g is generated from WDR82 intron.	Txn	N
miR-30b	*Sox9*	−0.6	Master transcription factor for chondrogenesis	Dev, Txn	N
miR-30b	*LIFR*	−0.6	Key factor for ES cell self-renewal. making heterodimer with gp130. Ligands are LIF and oncostain M. A member of IL-6 receptor family.	Stem, Dev, signals	M
miR-30b	*LRP6*	−0.5	Frizzled co-receptor for Wnt signaling	Dev, signal	M
miR-30b	*LIN28A*	−0.46	Inhibit pri-let-7 maturation in cytoplasm. Reprogramming factor. Containing CSD and CCHC ×2.	RNA, Stem	C
miR-30e	*LIN28B*	−0.71	Inhibit pri-let-7 maturation in nucleus. Reprogramming factor. Containing NoLS and NLS in addition to LIN28A.	RNA, stem	N
miR-30c	*S100PBP*	−1.19	Ca, Zn/transport		
miR-30c	*ZBTB41*	−0.92	Zinc finger and BTB domain containing 41	Txn	N
miR-30c	*CCNT2*	−0.49	Transcription, component of pTEFb with CDK9. phosphorylation of RNA polymerase II CTD for transcription elongation	Txn	Chro
miR-30c	*ZBTB44*	−0.47	Zinc finger and BTB domain containing 41	Txn	N
miR-30c	*CTGF/CCN2*	ref. 67	Regulates chondrocyte and osteoblast differentiation and angiogenesis. TGF inducible.	Dev	EC
miR-30d	*GRP78/HSPA5*	ref.	ER stress response	Chaperone	ER
miR-30e	*BRWD1*	−0.9	WD repeat domain. bromo domain recognize acetylated lysine in histone	Epige	N
miR-30e	*PCGF5*	−0.51	polycomb group (PcG) ring fnger 5	Epige	Chro
miR-30e	*HELZ*	−0.46	ZF RNA helicase	RNA	N

TargetScan was utilized for the prediction of targets and scoring. Listed factors are selected under score −0.4 and involved in development, RNA regulation, epigenetics and transcription. Abbreviations: Phos Sig, phosphorylation signal; Dev, development; Txn, transcription; RNA, RNA regulation; Epige, epigenetics; Stem, stem cell differentiation; C, cytoplasm; N, nucleoplasm; Chro, chromatin; EC, extracellular space; ER, endoplasmic reticulum.

Searching 3′-UTR of putative target mRNA, targeting sequences which can make base pairing with 5′ seed sequences of miR-30 were found in the 3′-UTR of *lifr, eed, pcgf5* and *sirt1* utilizing TargetScan (Fig 7B). One miR-30 targeting sequence in the 3′-UTR of *ctgf/ccn2* has been reported. In addition, two putative miR-30 targeting sites on *spp1/osteopontin* were found. Matching around the 3′ part and intermediate part of miR-30 were tested to those targets. Not only 5′ seed sequences but also 3′ sequences of miR-30d matched to the *lifr, eed* and *sirt1* 3′-UTR. On the other hands, miR-30b/c 5′ seed as well as 3′ part was matched with 3′-UTR sequences of *spp1/opn*, *pcgf5, hspa5/grp78 and ctgf/ccn2*. These *in silico* analyses suggested putative shared and distinctive target mRNA recognition by miR-30 family, the groups of miR-30a/d/e and miR-30b/c.

### miR-30 targeting in mMSC line

**Figure 8:**
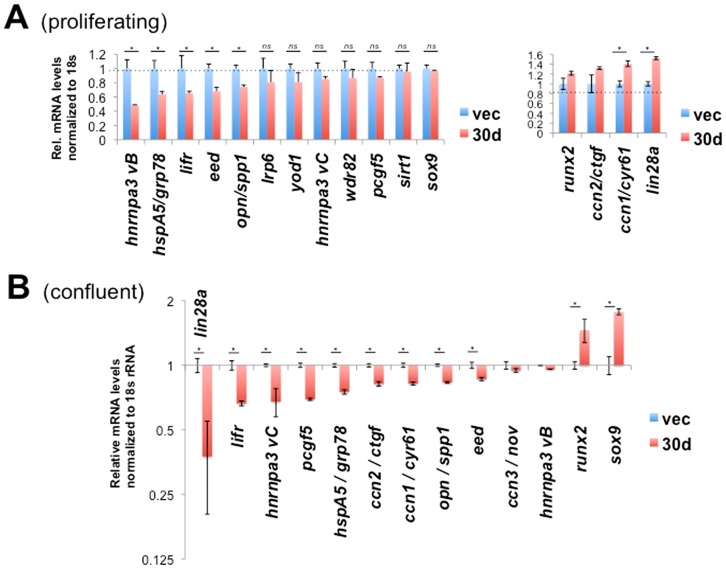
Analysis of miR-30 targeting. (A) Effects of miR-30d on mRNA levels in proliferating/sparse KUSA-A1 cells. Reduced and induced mRNA was separately shown. vec, vector transfectant; 30d, miR-30d transfectant. (B) Effects of miR-30d on target mRNA levels in confluent KUSA-A1 cells. *, *P*<0.05 (n = 4). *n.s.*, not significant.

In order to clarify the function of miR-30d on target mRNAs, qRT-PCR was carried out in stable miR-30d transfected KUSA-A1 and in control vector transfectant. In a result, *hnrnpa3 variant B* level in proliferating/sparse miR-30d tranfectant was around 50% lower than that in the vector transfected control (Fig 8A), while no significant change in confluent cells (Fig 8B), indicating context dependent repression of *hnrnpa3 vB* by miR-30d. *Lin28a* mRNA level in confluent miR-30d tranfectant was around 50% lower than that in the vector transfected control (Fig 8A, left), while around 50% higher in proliferating cells (Fig 8A, right), indicating context dependence as well. *Ccn2/ctgf* and *ccn1/cyr61* mRNA levels in confluent miR-30d cells were lower than those in the control (Fig 8B), while these gene product levels in proliferating miR-30d cells were higher than those in the control (Fig 8A). *Runx2* and *sox9* mRNA level in miR-30d transfectants were higher than that in the control (Fig 8AB). *Hspa5/grp78, lifr, eed, opn/spp1* and *pcgf5* mRNA levels in miR-30 transfected cells were 20–30% lower than those in control cells in both proliferating and confluent cells (Fig 8AB), indicating direct repression of mRNA stability. These data suggested targets of miR-30d and context-dependent effect of miR-30d on RNA regulators including lin28 and *hnRNPA3* and on differentiation regulators including *runx2, sox9 and ccn1/2*.

### Expression pattern of miR-30 targets

**Figure 9:**
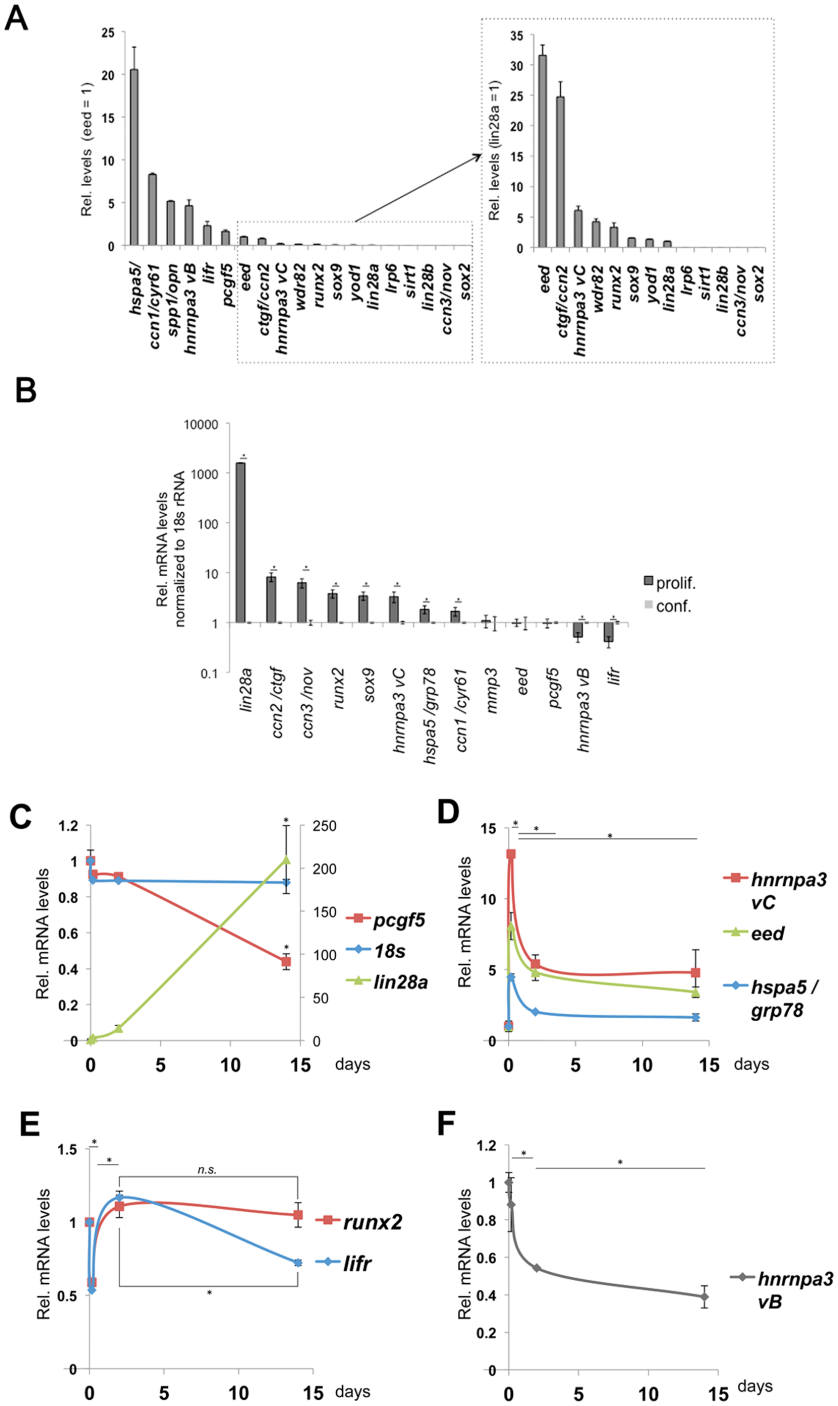
mRNA expression patterns of miR-30 targets in mMSC line. (A) Relative expression levels of miR-30 target mRNA in proliferating/sparse KUSA cells. Values were normalized to 18s rRNA levels. (B) Relative expression levels of miR-30 targets between proliferating and confluent cells. *, *P*<0.05 (n = 4). (C, D, E) Expression patterns of *lin28a, pcgf5* (C), *hnrnpa3 variant C, eed, hspa5/grp78* (D), *runx2*, *lifr* (E) and *hnrnpa3 variant B* (F) during KUSA-A1 osteocytogenesis. *, *P*<0.05 (n = 2). *n.s.*, not significant.

For a better understanding of miR-30 targeting, basal mRNA expression levels of 18 gene products were quantified and compared in proliferating/sparse KUSA-A1 cells (vector transfected control cells). *HspA5/grp78, ccn1/cyr61, spp1/opn, hnrnpa3 vB, lifr, pcgf5, eed* and *ctgf/ccn2* were detected in high-level expression levels (Fig 9A). *Hnrnpa3 vC*, *wdr82, runx2, sox9, yod1* and *lin28a* were detectable levels and quantitated. The expression levels of *lrp6*, *sirt1* and *lin28b* were quite low. The mRNA levels of *ccn3/nov* and *sox2* were under detection limit (Fig 9A). Interestingly, RNA regulators (*lin28a, hnrnpa3 vC*) and differentiation-related factors (*ccn2, ccn3, runx2, sox9*) were expressed in proliferating KUSA-A1 cells, while suppressed in confluent cells (Fig 9B). These mRNA including *runx2*, *ccn2/ctgf, ccn3/nov* and *opn/spp1* (Fig 2) were again induced upon osteo-induction (Fig 2). Oppositely, *hnrnpa3 vC* and *lifr* mRNA levels increased in confluency compared with those in proliferating cells (Fig 9B). These results suggest that many gene products encoding growth factors and transcription factors were powerfully expressed in proliferating cells compared with idling cells, while only *lifr* and *hnrnpa3 vC* mRNA are increased in idling confluent cells compared with proliferating cells.

Furthermore, mRNA expression pattern of miR-30d targets during osteogenesis of KUSA cells were quantified. *Lin28a* mRNA was remarkably induced during the osteogenesis, and reached around 200-fold on day 14 (osteocytic stage) compared with the day 0 (Fig 9C), indicating its essential role and possible release of repression during osteogenesis. *Pcgf5* mRNA levels decreased, and reached to the half on day 14 compared with the level on day 0 (Fig 9C). *Hnrnpa3* variant c, *eed* and *hspa5* mRNA were immediately induced by the osteo-inductive stimulation by 13-fold, 8.1-fold and 4.4-fold respectively, and thereafter kept around half levels of the maximum levels (Fig 9D). These immediate early induction followed by quick attenuation patterns were shared with those of *CCN* gene family shown in Fig 2A, indicating these 6 kinds of transcripts are under the control of same factors and the miR-30 family. Distinctively from the variant C, *hnrnpa3 variant B*, the major variant, mRNA was gradually reduced upon osteocytogenesis (Fig 9F), indicating osteo-induction controls splicing of *hnRNPA3* mRNA. Both *runx2* and *lifr* mRNA were immediately reduced at 4 hours after the stimulation, and thereafter recovered in 2 days (Fig 9E). Then *lifr* mRNA level was reduced to the day 14, while *runx2* mRNA did not decrease and kept the expression level comparable to the initial level. Together with the data of expression patterns in Fig 9 and Fig 2, miR-30 targets were classified into several groups; immediate induction followed by rapid attenuation group (*ccn1/2/3, hnrnpa3 vC, eed, hspa5/grp78*), immediate reduction and rapid recovery group (*runx2 and lifr*), the constant induction group (*lin28a* and *opn/spp1*) and the constant reduction group (*pcgf5 and hnrnpa3 vB*).

### Human miR-541 and miR-155 function and expression pattern in hMSC/hMBSC osteoblastic differentiation

**Figure 10:**
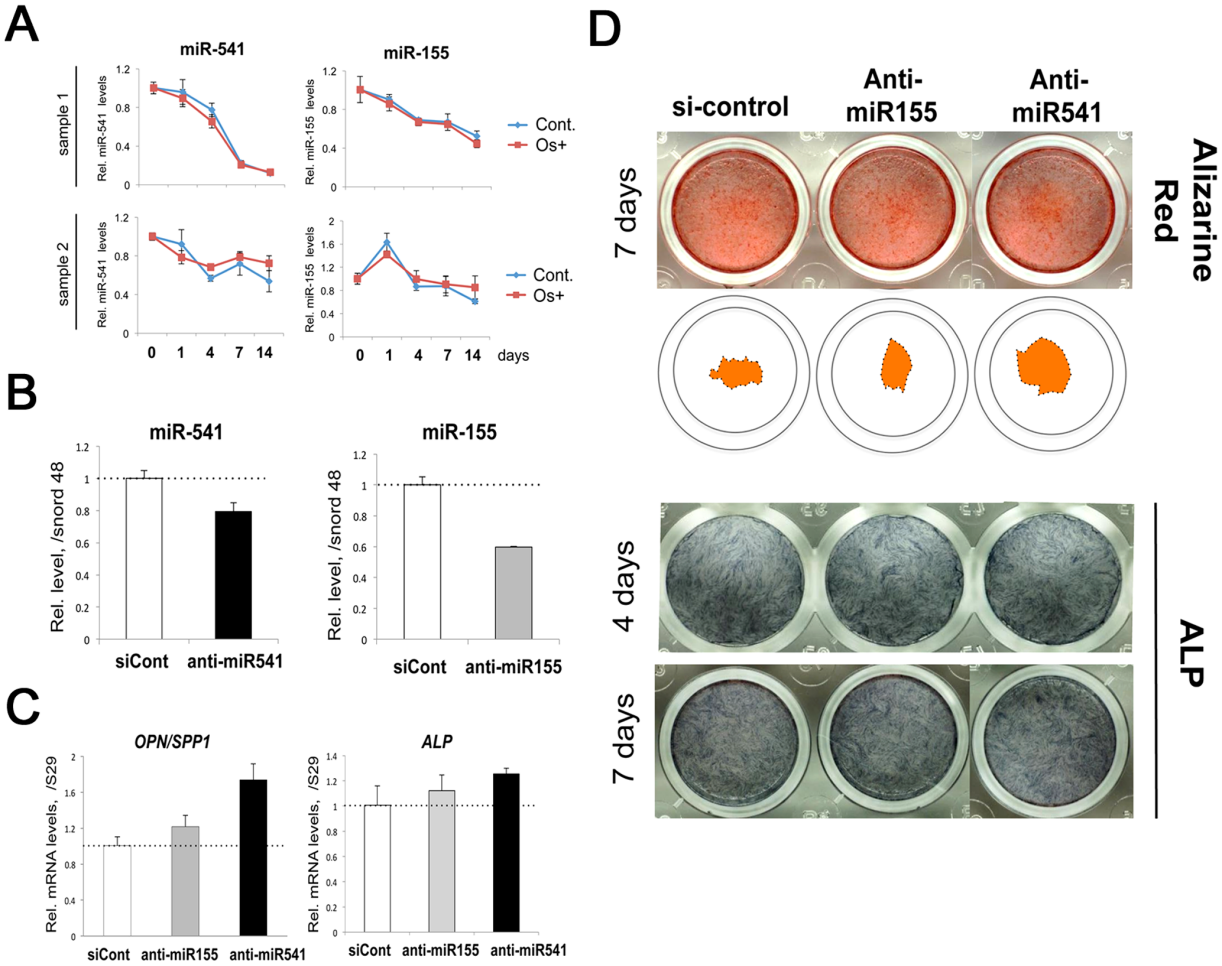
Expression and function of miR-541 and miR-155 during hBMSC/MSC osteogenesis. (A) Expression pattern of miR-541 and miR-155 with (osteo) or w/o (control) osteo-induction. (B) Knock-down of miR-541 and miR-155. miRNA levels were normalized to Snord48 levels. (C) Effect of anti-miR-541 or anti-miR-155 on *OPN/SPP1* and *ALP* mRNA expression. Values were normalized to S29 mRNA levels. (D) Effect of anti-miR-541 and anti-miR155 on calcification (top row) and alkaline phosphatase production (ALP, bottom 2 rows). The thick stained part in the center by alizarin red S was re-organized in the 2nd row.

In order to investigate OstemiR expression in hMSC osteoblastic differentiation, qRT-PCR was carried out. miR-541 and miR-155 were induced in 4 hours after the osteo-stimulation to KUSA-A1, but not on the day 14 (Fig 3B, Fig 4). In addition, miR-155 and miR-541 were gradually reduced during osteoblast differentiation of hMSC (Fig 1H), while miR-541 level was higher in osteo-induced cells on day 4 and day 14 compared with the control (Fig 10A). In attempt to clarify a function of miR-541 and miR-155 during osteogenesis of hMSC, we transiently transfected these cells with antagonists/anti-miR targeting these two types of miRNAs. The efficiency of anti-miR-155 or anti-miR-541 knockdown was of approximately 20–40% compared with the control siRNA transfections (Fig 10B). Despite this apparently low efficiency, a significant increase by 1.8-fold in the osteoblastic marker *OPN/SPP1* mRNA level was observed by miR-541 knock down (Fig 10C). *ALP* mRNA level in anti-miR-541 transfectants was higher than that in the control. In accordance, calcium deposition on the anti-miR541-treated cells was more rich than those of controls in a result of alizarin red S staining on day 7, comparing center regions in each wells (Fig 10D). There were no significant changes in ALP staining of upon miR-155 or miR-541 knock down. Taken together, these data indicate that miR-541 is a negative regulator of osteoblast differentiation of hMSC.

## Discussion

### Tuning model of osteogenic factors by OstemiRs during MSC osteogenesis

**Figure 11:**
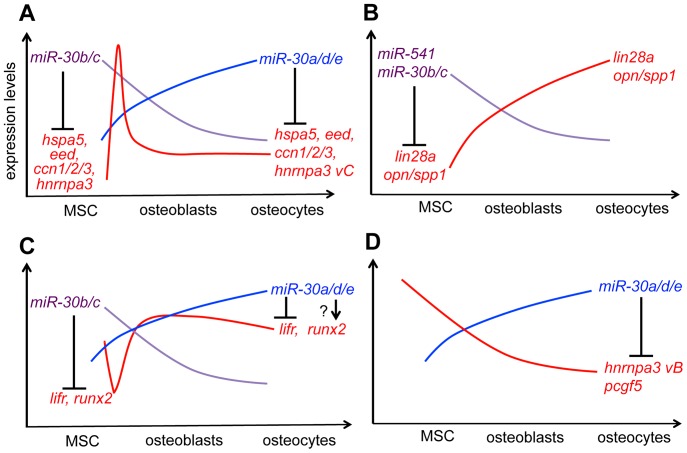
Tuning model of canonical and novel osteogenic factors by miRNA-30 family and miR-541 during MSC osteogenesis. miR-30b/c repress *hspa5, eed, ccn1/2/3, hnrnpa3 vC* (A), *lin28a, opn/spp1* (B), *lifr* and *runx2* (C) in MSC stage. This repression is released during osteogenesis due to reduction of miR-30b/c, especially significantly in increase in *opn/spp1, lin28a* (B), *lifr* and *runx2* (C). Osteo-induction transiently induces *hspa5, eed, ccn1/2/3* and *hnrnpa3 vC*, thereafter those transcripts are attenuated by miR-30b/c in early stage and by miR-30a/d/e in osteocytic stage (A). miR-30a/d/e target *hnrnpa3 vB* and *pcgf5* in osteoblastic and osteocytic stages (D). Human *OPN/SPP1* in hBMSC/MSC is attenuated by miR-541.

Together with these results and data interpretations, we propose the tuning model of canonical and novel osteogenic factors by the OstemiRs including miR-30 family and miR-541. In this model, miR-30b/c represses *hspa5, eed, ccn1/2/3, hnrnpa3 vC* (Fig 11A), *opn/spp1, lin28a* (Fig 11B), *lifr* and *runx2* (Fig 11C) at the MSC stage. This repression is released during osteogenesis upon reduction of miR-30b/c, a change especially significantly in increase in *opn/spp1, lin28a* (Fig 11B), *lifr* and *runx2* (Fig 11C). Osteo-inductive stimulation transiently induces *hspa5, eed, ccn1/2/3* and *hnrnpa3 vC*, but thereafter those transcripts are attenuated by miR-30b/c at the early stage and by miR-30a/d/e during the osteocytic stage (Fig 11A). miR-30a/d/e targets *pcgf5 and hnrnpa3 vB* as well (Fig 11D). Human *OPN/SPP1* in hBMSC/MSC is attenuated by miR-541.

### Osteogenic differentiation of an mMSC line and hMSCs

The speed of mineralization was different among the cell types tested: KUSA>hMSC>MC3T3 as shown in Fig 1. In addition, Comparing expression patterns of opn/spp1 between KUSA and hMSC/hBMSC, it was suggested that KUSA-A1 is most committed to osteoblast lineage, or has a faster differentiation process than hBMSC, which would contain more undifferentiated cells and differentiation potential to other lineage. Thus, further understanding and sorting MSC and BMSC are needed for clinical application for bone regeneration, because those populations have potentials of aging/senescence, adipogenic and chondrogenic differentiation as well.

The difference in speed or stage of differentiation may result in difference in expression signature of miRNA, e.g., mouse miR-30d was induced on 4 h or 14 days after the osteo-induction compared with the control, while human miR-30d showed waving induction and reduction during osteogenesis (Fig S2). Besides, mouse miR-541 was strongly induced in 4 hours of initial induction, and then reduced in the later stage (Fig 3B and Fig 4), while in hMSC, miR-541 was gradually reduced in long culture, suggesting some role for miR-541 in osteogenesis. In fact, knockdown of miR-541 upregulated *OPN/SPP1* and mineralization. However, a direct target site of miR-541 on *OPN/SPP1* has not been identified yet, indicating an unknown indirect mechanism.

### Putative roles of novel key factors in osteogenesis: Lin28A, hnRNPA3, Eed and Pcgf5

As targets of miR-30, we found novel key factors in osteogenesis including Lin28, hnRNPA3, Eed, Pcgf5 and HspA5/Grp78. Here we discuss about roles of these factors in bone formation as well as canonical osteogenic factors including Runx2, LifR, Opn/Spp1 and the CCN family, which are the targets of miR-30d.

LIN28A is essential in induced pluripotent stem cells (iPSC) and represses the let-7 tumor-suppressor miRNA family [40]. A recent study proposed that *Lin28* is essential in embryonic stem cells (ESC), induced pluripotent stem cells (iPSC) and tumorigenesis and that the expression of *LIN28* is controled by let-7, miR-9, miR-125 and miR-30 [41], indicating not only miR-30, but let-7, miR-9 and miR-125 can control lin28a during osteogenesis.

Heterogeneous nuclear ribonucleoprotein (hnRNP) is another crucial RNA regulator in RNA nuclear export and splicing. hnRNPA1 directly associates with miR-18a stem-loop as well as pri-miR-17/18a/19a, and then export pri-miR-17/18a/19a in the exportin-independent manner [42]. Thereafter, hnRNPA1 promotes cleavage of the miRNA by Drosha and Dicer. Furthermore, hnRNPA1 inhibits processing of pri-let-7a by competing to KSRP, which promote Drosha processing let-7a [43]. Nuclear export of hnRNPA1 is promoted by phosphorylation in cells stressed by osmic shock [44]. The role of hnRNPA3 is presumably similar to that in hnRNPA1. In our study, only *hnrnpa3 variant C* was induced upon osteo-induction, but not variant B, and context-dependent effect of miR-30d on hnRNPA3 variants was suggested. Thus, context- or osteoinduction-dependent hnRNP variants presumably control mRNA splicing as well as the processing of miRNAs.

EED, named after embryonic ectoderm development, is another novel target of miR-30. Eed is one of the main components of polycomb repressive complex 2 (PRC2), which induces histone H3K9 and K27 methylation leading to gene repression. Recent studies revealed crucial roles of Eed in ESC self-renewal through interaction with Stat3, Oct-3/4 and Sox2 [45], [46]. Eed was also induced during osteo-induction in our study. However, sox2, an ESC marker as well as an iPSC inducer, was undetected in KUSA cells. Therefore, MSC was distinguished from ESC or iPSC in this point, although our data suggested that Eed-mediated silencing through histone methylation has still some role in osteogenesis. PCGF5, a polycomb group ring finger protein is involved in gene repression as well. This reduction of polycomb factor PCGF5 during the osteogenesis may release expression of osteocytogenic factors and miRNAs in an epigenetic manner.

### miR-30 controls expression of LifR and Runx2, the known regulators for osteoblasts

LIFR, the leukemia inhibitory factor receptor, is essential in ESC self-renewal and in bone marrow stromal osteoblast differentiation. Under physiological conditions, LIF is produced from articular and growth plate cartilage, promoting proliferation and differentiation of chondrocytes [47]. LIF from chondrocytes influences vascularization during bone growth through promotion of chondroclasts and osteoclasts. LIF produced in the marrow, and by osteoblasts on the bone surface acts on stromal precursors to inhibit adipogenesis, and stimulates osteoblasts on bone remodeling surfaces. LIF also acts on osteocytes to inhibit production of SOST/sclerostin. In pathology, LIF released by inflamed synovium contributes to cartilage destruction by altering MMP production, and is likely to induce osteoclastic bone erosions in rheumatoid arthritis. As observed in Fig 11C, suppression of *lifr* expression by miR-30 may control osteoblast and osteocyte differentiation leading to attenuation of Lif/LifR/Jak-Stat signal.

Runt-related transcription factor 2 (Runx2) is a master transcription factor for osteoblast/osteocyte differentiation and is also known as core-binding factor subunit alpha-1 (CBFA1) and acute myeloid leukemia 3 (AML3). Mutations in Runx2/Cbfa1 are associated with Cleidocranial dysplasia, which causes underdevelopment of bones and joints and multiple unerupted supernumerary teeth. A previous study showed that runx2 is a target of miR-30c, miR-135a, miR-204, miR-133a, miR-217, miR-205, miR-34, miR-23a and miR-338 [34]. Our data also indicate that miR-30b/c represses *runx2* mRNA; however, overexpression of miR-30d increased *runx2* expression, through unknown mechanisms. *Runx2* and *osterix* are essential for osteoblastic differentiation. However, transcription factors that regulate osteoblast maturation and/or osteocytic differentiation in late or terminal differentiation stages have not yet been found. Thus, a cohort of the OstemiR could be a crucial regulator of terminal differentiation in the osteoblast lineage. Although *runx2* is essential for osteocytic differentiation, *runx2* transgenic mice showed loss of bone mass and osteocytes, suggesting that *runx2* represses the late stages of osteocytic differentiation [48]. By contrast, *osterix/sp7* is highly expressed in osteocytes, and conditional knockout of osterix in mice resulted in a disorder of osteocytes and osterix target gene expression [49]. It has been hypothesized that osteocytes have a metabolostat role for homeostasis in bone [50]. Together with these findings, not only vast expression of master transcription factors but spacio-temporal diligent control by OstemiR would contribute to development and homeostasis in bone. In order to understand and control bone formation, other transcription factors essential for osteoblast and osteocyte differentiation, e.g., Osterix/Sp7 [51], Dlx3/Dlx5 [52], [53] and Mef2c [31], [54] as well as crucial markers/factors for osteocytogenesis e.g. Sost/sclerostin, PTH1R, FGF23, Phex, MEPE [55], Dmp and InsR/insulin signal [56]. Further investigation of OstemiR targeting on these osteogenic factors is underway.

### miR-30 controls *CCN* family gene expression during MSC osteogenesis

Physiological production of CCN2/CTGF is more abundant from chondrocytes in cartilage than those in other tissues, while CCN1/2/3, the prototype members of CCN family, control both chondrocytic and osteoblastic differentiation [57, 58). The transient induction of *ccn1/2/3* observed in this study could be necessary and essential in initiation of MSC differentiation. A *cis*-acting element of structure-anchored post-transcriptional repression (CAESAR) was identified in the 3′-UTR of *ccn2* in human [59], [60], [61], then similar elements was found in mouse [60] and in chicken [62], [63]. In addition, miR-26ab/1297, miR-132/212, miR-133, miR-18ab and miR-19, members of OstemiR, were predicted to recognize the 3′-UTR of *ccn2/ctgf*. Among this group, miR-18 has been reported to control *ctgf/ccn2* gene expression in chondrocytic cells [64]. Dexamethasone has been shown to be a strong inducer of *ctgf/ccn2* [65], [66]. In our study, *ctgf/ccn2* was immediately triggered after the osteo-induction treatment that included dexamethasone, and then rapidly repressed in 2 days. Therefore, immediate induction and subsequent rapid repression of *ctgf/ccn2* could be controlled by fluctuations in these miRNAs including the miR-30 family. In myocardial cells CTGF/CCN2 is regulated by miR-133 and miR-30c [67] and the 3′-UTR of *ctgf* and miR-30c are basepairing by 9 bases at 5′ seed of miR-30c and 11 bases at 3′ part including one of each GU non-Watson-Crick base pairing (Fig 7A). In a result of direct analysis of *ctgf/ccn2* mRNA, miR-30d reduced ctgf/ccn2 mRNA levels in confluent KUSA-A1, while not in proliferating cells (Fig 8), indicating that miR-30d attenuate basal *ctgf/ccn2* level in idling MSCs.

### Possible application of OstemiR for osteoporosis, periodontitis and peri-implantitis

The OstemiR revealed in this study will be further clarified in studies aimed at understanding and controlling distinct regulation of MSC differentiation into not only osteocyte but also adipocytes, chondrocytes, myoblasts and tendon/ligament cells. In fact, *runx2* as well as *sox9* a master transcription factor for chondrogenesis was upregulated in mRNA level by miR-30d, indicating miR-30 could direct differentiation of MSC. We focused on the miR-30 family and miR-541 in this study, while still further analyzing roles of OstemiR in MSC differentiation. Kawashima et al. analysed the gene expression signature of KUSA-A1 cells using cDNA microarray [33]. Comparing the protein coding gene and miRNA expression signatures in future investigations could potentially clarify target mRNAs of OstemiR. Combination of several OstemiR or anti-OstemiR might regulate firmer cell differentiation. miRNAs have the advantage of being smaller molecules than proteins or antibodies and more easily synthesized or quantified compared to the other molecules. Moreover, recent studies have demonstrated that many miRNAs can be secreted [10]. Application of OstemiRs could be useful for treatment of bone-related diseases such as periodontitis, peri-implantitis and osteoporosis as well as diagnosis.

## Supporting Information

Figure S1 **Prediction of miRNA recognition sequences in the 3′UTR of human DMP1.**
(PDF)

Figure S2 **Prediction of miRNA recognition sequences in the 3′UTR of human Runx2/Cbfa1.**
(PDF)

Figure S3 **Prediction of miRNA recognition sequences in the 3′UTR of human Nov/CCN3.**
(PDF)

Figure S4 **Prediction of miRNA recognition sequences in the 3′UTR of human CTGF/CCN2.**
(PDF)

Tables S1 (DOCX)
