# Supplementary figures and images for: OstemiR: A Novel Panel of MicroRNA Biomarkers in Osteoblastic and Osteocytic Differentiation from Mesencymal Stem Cells
Source: PLoS One. 2013 Mar 22;8(3):e58796. doi: 10.1371/journal.pone.0058796 (PMC3606401; doi:10.1371/journal.pone.0058796)

## Human DMP1 3' UTR

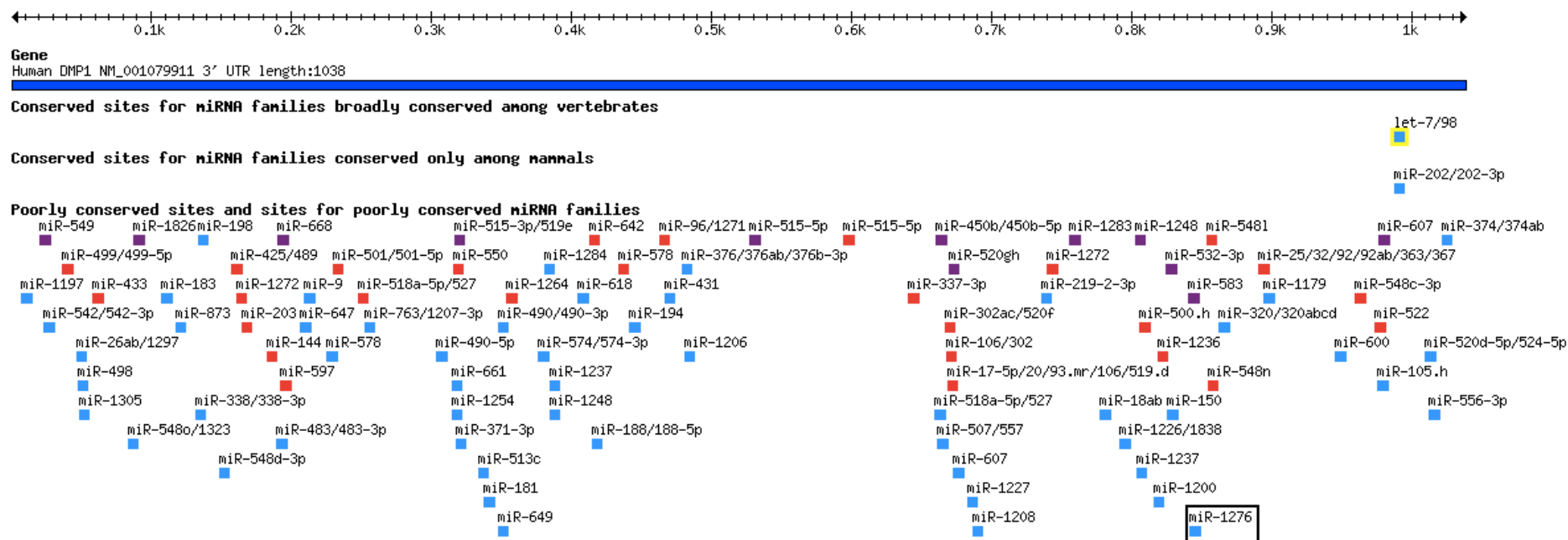

**Fig S1**

Supplement: Figure S1 — Prediction of miRNA recognition sequences in the 3′UTR of human DMP1. (PDF) [file pone.0058796.s001.pdf]

# Human RUNX2 3' UTR

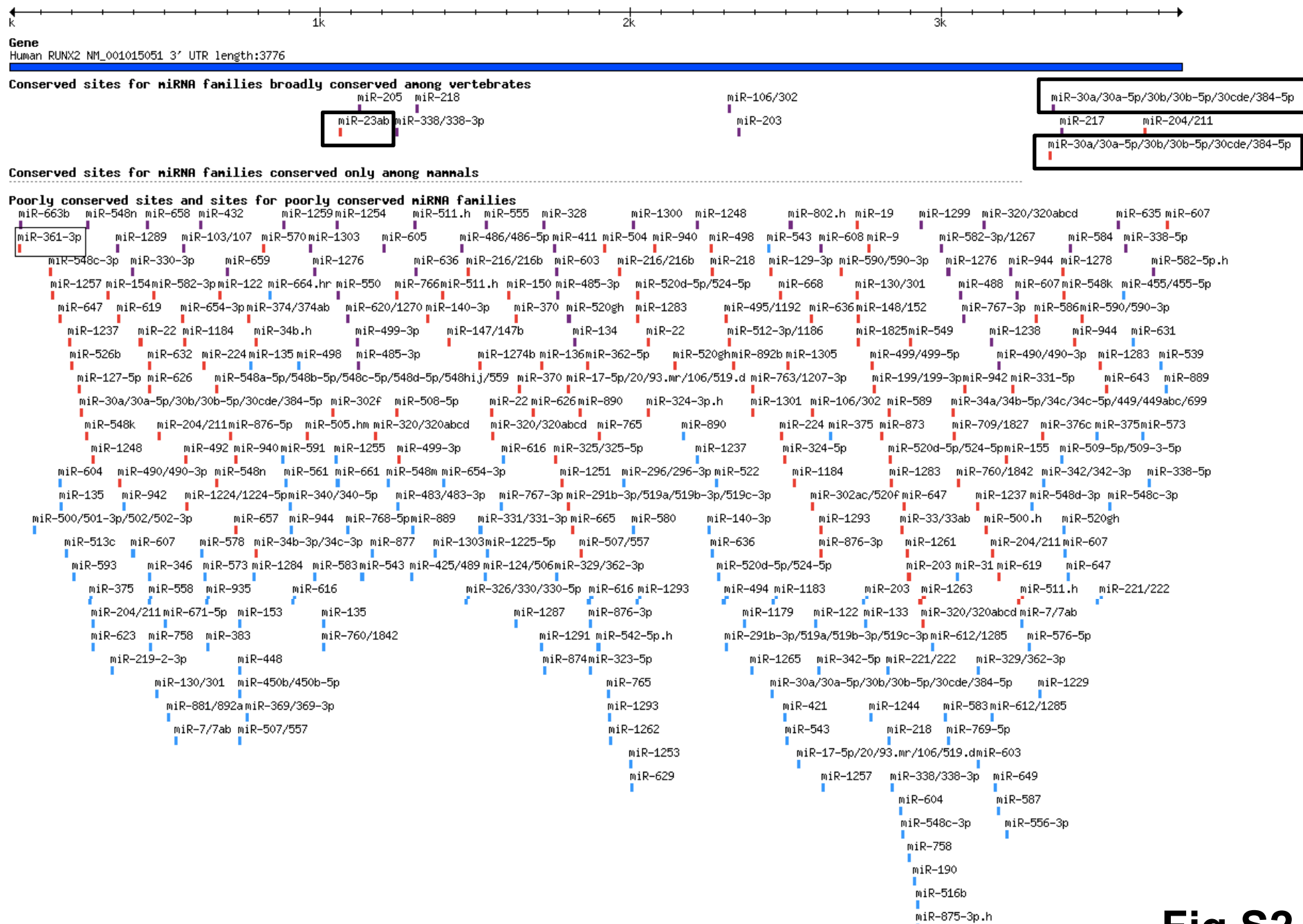

**Fig S2**

Supplement: Figure S2 — Prediction of miRNA recognition sequences in the 3′UTR of human Runx2/Cbfa1. (PDF) [file pone.0058796.s002.pdf]

## Human NOV 3' UTR (CCN3)

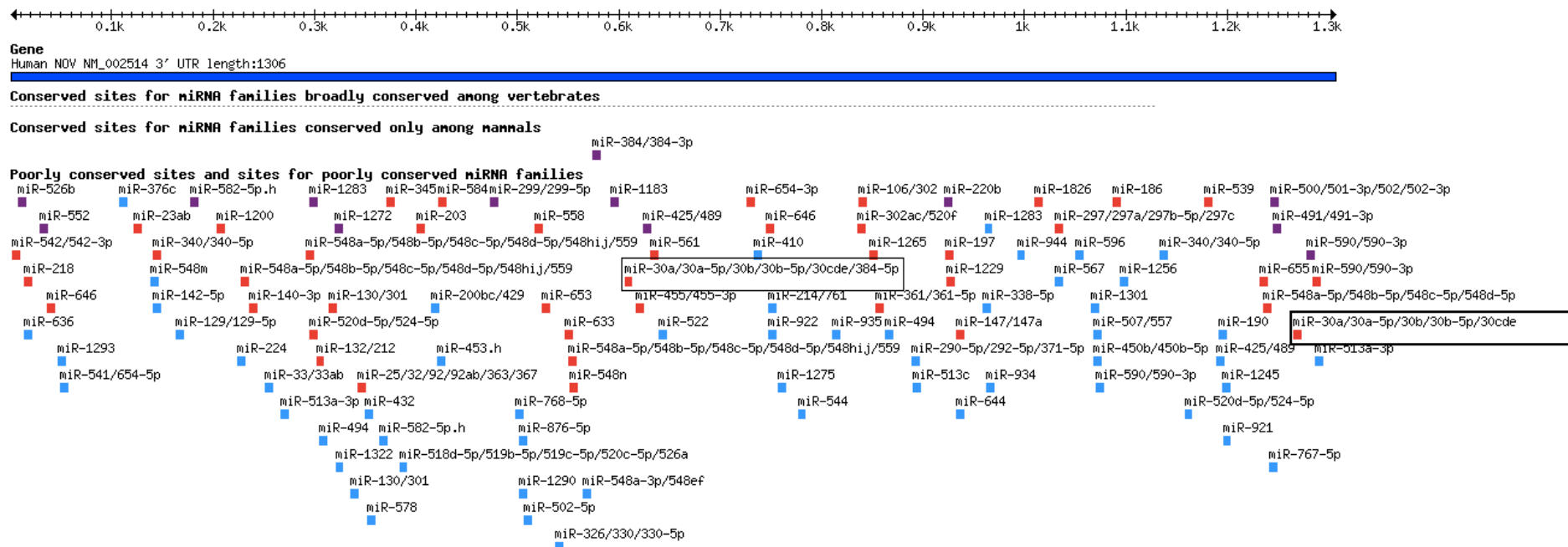

Fig S3

Supplement: Figure S3 — Prediction of miRNA recognition sequences in the 3′UTR of human Nov/CCN3. (PDF) [file pone.0058796.s003.pdf]

## Human CTGF 3' UTR (CCN2)

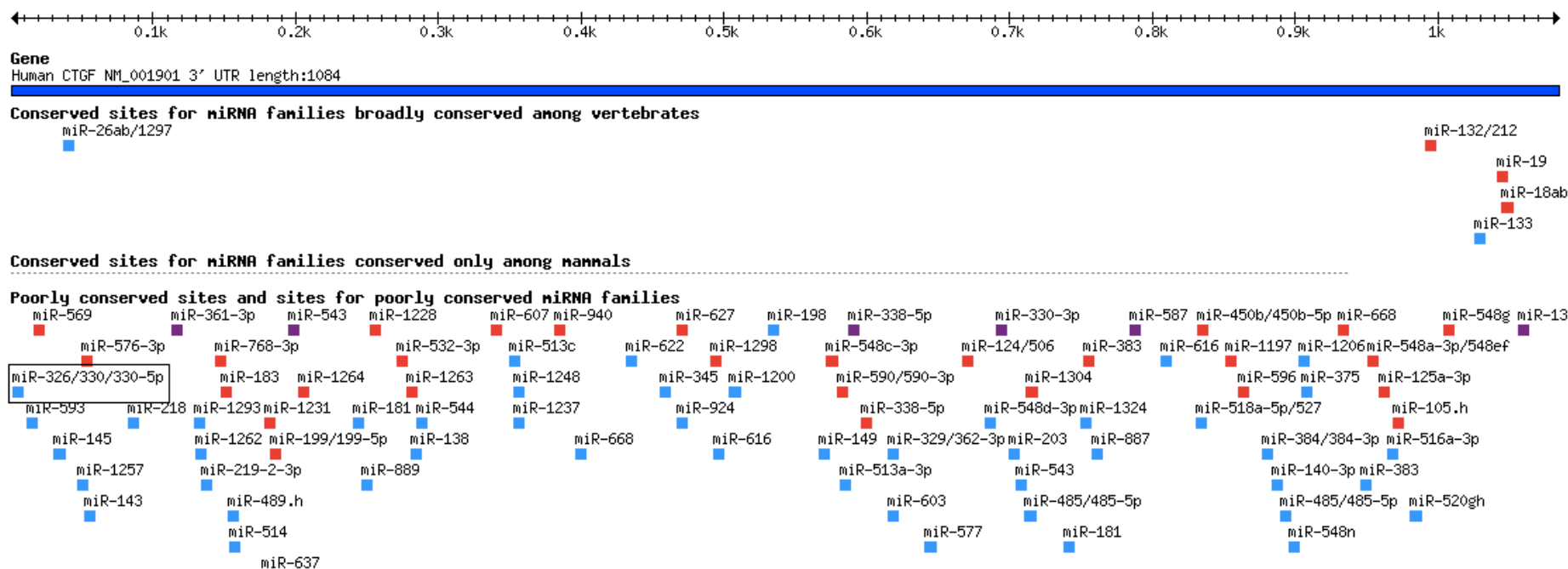

**Fig S4**

Supplement: Figure S4 — Prediction of miRNA recognition sequences in the 3′UTR of human CTGF/CCN2. (PDF) [file pone.0058796.s004.pdf]
