# Supplementary material for: OstemiR: A Novel Panel of MicroRNA Biomarkers in Osteoblastic and Osteocytic Differentiation from Mesencymal Stem Cells
Source: PLoS One. 2013 Mar 22;8(3):e58796. doi: 10.1371/journal.pone.0058796 (PMC3606401; doi:10.1371/journal.pone.0058796)
Supplement: Tables S1 — (DOCX) [file pone.0058796.s005.docx]

| primer name | sequence, 5' to 3' | GenBank ID | cDNA size | position | intron size | variant cover | product size |
| --- | --- | --- | --- | --- | --- | --- | --- |
| [Lin28A mF](http://www.ncbi.nlm.nih.gov/sites/entrez?Db=gene&Cmd=ShowDetailView&TermToSearch=79727) | gtctggaatccatccgtgtc | [NM_145833.1](http://www.ncbi.nlm.nih.gov/sites/entrez?cmd=search&db=nucleotide&dopt=GenBank&term=NM_145833.1) | 3480 | 441 | 1589 |  | 100 |
| [Lin28A mR](http://www.ncbi.nlm.nih.gov/sites/entrez?Db=gene&Cmd=ShowDetailView&TermToSearch=79727) | cctttggatcttcgcttctg |  |  |  |  |  |  |
| Lin28B mF | tgtgcgagaagaagagtcca | [NM_001031772.2](http://www.ncbi.nlm.nih.gov/sites/entrez?cmd=search&db=nucleotide&dopt=GenBank&term=NM_001031772.2) | 5420 | 621 | 37705 | 1/2 | 103 |
| Lin28B mR | gcacttctttggctgaggag |  |  |  |  |  |  |
| Eed mF | cacaaatacgccaaatgcac | [NM_021876.3](http://www.ncbi.nlm.nih.gov/sites/entrez?cmd=search&db=nucleotide&dopt=GenBank&term=NM_021876.3) | 2060 | 687 | 4573 |  | 129 |
| Eed mR | actccaaacaatggctggtt |  |  |  |  |  |  |
| Pcgf5 mF | gacggaatgcctccatacat | [NM_029508.3](http://www.ncbi.nlm.nih.gov/sites/entrez?cmd=search&db=nucleotide&dopt=GenBank&term=NM_029508.3) | 1509 | 394 | 22333 |  | 92 |
| Pcgf5 mR | catgaacttggttgccacac |  |  |  |  |  |  |
| Lifr mF | ttcaaacagcacggagactg | [NM_001113386.1](http://www.ncbi.nlm.nih.gov/sites/entrez?cmd=search&db=nucleotide&dopt=GenBank&term=NM_001113386.1) | 2844 | 2478 | 2684 | 2/2 | 90 |
| Lifr mR | ccctggttagtgcacccata |  |  |  |  |  |  |
| Lrp6 mF | ggattattgtccccggatg | [NM_008514.4](http://www.ncbi.nlm.nih.gov/sites/entrez?cmd=search&db=nucleotide&dopt=GenBank&term=NM_008514.4) | 9368 | 2008 | 521 |  | 80 |
| Lrp6 mR | atacgattagtttcagaatctgtcca |  |  |  |  |  |  |
| hnRNPA3 vc mF | gctatttctgctgccctgaa | [NM_053263.1](http://www.ncbi.nlm.nih.gov/sites/entrez?cmd=search&db=nucleotide&dopt=GenBank&term=NM_053263.1) | 5176 | 4472 | - | 3/3 | 88 |
| hnRNPA3 vc mR | gcagatttgtcccctgaaaa |  |  |  |  |  |  |
| hnRNPA3 vb mF | gctatttctgctgccctgaa | [NM_146130.3](http://www.ncbi.nlm.nih.gov/sites/entrez?cmd=search&db=nucleotide&dopt=GenBank&term=NM_146130.3) | 5242 | 4538 | - | 3/3 | 110 |
| hnRNPA3 vb mR | ttccctgtgtgggaaacatt |  |  |  |  |  |  |
| wdr82 mF | tgcaattcaaccccaagttt | [NM_029896.1](http://www.ncbi.nlm.nih.gov/sites/entrez?cmd=search&db=nucleotide&dopt=GenBank&term=NM_029896.1) | 4313 | 1186 | 2083 |  | 102 |
| wdr82 mR | aaatagcccagcagcatcag |  |  |  |  |  |  |
| grp74/hspA5 mF | cagatcttctccacggcttc | [NM_001163434.1](http://www.ncbi.nlm.nih.gov/sites/entrez?cmd=search&db=nucleotide&dopt=GenBank&term=NM_001163434.1) | 2534 | 1540 | 317 | 2/2 | 126 |
| grp74/hspA5 mR | agcaggaggaattccagtca |  |  |  |  |  |  |
| Sirt1 mF | gtctcctgtgggattcctga | [NM_001159589.1](http://www.ncbi.nlm.nih.gov/sites/entrez?cmd=search&db=nucleotide&dopt=GenBank&term=NM_001159589.1) | 3806 | 895 | 5558 | 2/3 | 97 |
| Sirt1 mR | caaacatggcttgagggtct |  |  |  |  |  |  |

Table S1. List of primers for qRT-PCR of miR-30 targets.

| Name of primer | GenBank # | sequences, 5' to 3' | position |
| --- | --- | --- | --- |
| mDMP1-QF | NM_016779 | AAGAACATGGAAGCTGACAGTAG | 1531-1553 |
| mDMP1-QR |  | GAGAGCCATTTCTTAGACAAGCTA | 1656-1633 |
| mSOST-QF | NM_024449 | CTGAGAACAACCAGACCATGAACC | 185-208 |
| mSOST-QR |  | GTCTGTCAGGAAGCGGGTGTAGT | 309-287 |
| mMEF2C-QF | NM_025282 | TTTCCGTAGCAACTCCTACTTTAC | 1274-1297 |
| mMEF2C-QR |  | AGAGATGACAGATCTGCGCTAC | 1385-1364 |
| mPhex-QF | NM_011077 | ATGATAGAAGACAGGGAGTTGAAG | 2515-2538 |
| mPhex-QR |  | TGGTCTATAGGAATTGCACCTTAC | 2624-2601 |
| mFGF23-QF | NM_022657 | TACTTGTCGCAGAAGCATCAC | 507-527 |
| mFGF23-QR |  | GTGGGCGAACAGTGTAGAAAT | 651-631 |
| mSpp1/Opn-QF | NM_009263 | GATGAATCTGACGAATCTCACC | 412-433 |
| mSpp1/Opn-QR |  | TCAGTCCATAAGCCAAGCTATC | 550-529 |
| mAlpl-Akp2-QF | NM_007431 | ACAAGCATTCCCACTATGTCTG | 1024-1045 |
| mAlpl-Akp2-QR |  | GTTCCGATTCAATTCATACTGC | 1142-1121 |
| mSp7/Osx-QF | NM_130458 | CTACTTACCCATCTGACTTTGCTC | 615-638 |
| mSp7/Osx-QR |  | TTATAGACATCTTGGGGTAGGACA | 717-694 |

Table S2. List of primers for qRT-PCR of osteocyte markers.

| Name of primer | sequences, 5' to 3' | product size |
| --- | --- | --- |
| mCCN1/Cyr61 F | ATG AAG ACA GCA TTA AGG ACT C |  |
| mCCN1/Cyr61 R | TGC AGA GGG TTG AAA AGA AC |  |
| mCCN2/Ctgf F | CCA CCC GAG TTA CCA ATG AC | 169 |
| mCCN2/Ctgf R | GTG CAG CCA GAA AGC TCA |  |
| mCCN3/Nov F | TGA AGT CTC TGA CTC CAG CAT T | 230 |
| mCCN3/Nov R | TGG CTT TCA GGG ATT TCT TG |  |
| mOcn/Bglap F | 5′-AAGCAGGAGGGCAATAAGGT-3′ | 156 |
| mOcn/Bglap R | 5′-TTTGTAGGCGGTCTTCAAGC-3′ |  |
| BSP mF | 5′-CACACTTTCCACACTCTCG-3′ | 122 |
| BSP mR | 5′-CCCTGCTTTCTGCATCTC-3′ |  |
| Runx2/AML3/Cbfa1 mF | 5′-ATT ACAGATCCCAGGCAGGCA-3′ | 176 |
| Runx2/AML3/Cbfa1 mR | 5′-CAGAAGTCAGAGGTGGCAGTGT |  |
| sox9 mF | 5-AGGCCACGGAACAGACTCA-3 | 170 |
| sox9 mF | 5-AGCTTGCACGTCGGTTTTG-3 |  |
| gapdh F | 5-ATCTTGGGCTACACTGAGGA-3 |  |
| gapdh R | 5-CAGGAAATGAGCTTGACAAAGT-3 |  |
| ribosomal S18 h/m/r F | 5’-GCGAATTCCTGCCAGTAGCATATGCTG-3’ |  |
| ribosomal S18 h/m/r R | 5’-GGAAGCTTAGAGGAGCAGCGACCAAAGC-3’ |  |
| mMmp3f | TGGAACAGTCTTGGCTCATGCCTA |  |
| mMmp3r | TGGGTACATCAGAGCTTCAGCCTT |  |

Table S3. List of primers for qRT-PCR.

|  | GenBank ID | cDNA length | sequence, 5' to 3' | Position | Product size | intron size |
| --- | --- | --- | --- | --- | --- | --- |
| RPs29 hF | BC032813 | 283 | TCTCGCTCTTGTCGTGTCTGTTC | 57 | 75 |  |
| RPs29 hR |  |  | ACACTGGCGGCACATATTGAGG | 131 |  |  |
| ALP hF | NM_000478 | 2,596 | GCACCGCCACCGCCTACC | 592 | 150 |  |
| ALP hR |  |  | CCACAGATTTCCCAGCGTCCTTG | 741 |  |  |
| OPN/SPP1 hF | BC007016 | 1,486 | ATGTGATTGATAGTCAGGAACTT | 835 | 89 |  |
| OPN/SPP1 hR |  |  | GTCTACAACCAGCATATCTTCA | 923 |  |  |
| SOST hF | [NM_025237.2](http://www.ncbi.nlm.nih.gov/sites/entrez?cmd=search&db=nucleotide&dopt=GenBank&term=NM_025237.2) | 2322 | ttgattcagtgccaaggtca | around 1440 | 137 |  |
| SOST hR |  |  | caggagtttgtcagccgtaa |  |  |  |
| hDMP1-f112 | [NM_001079911.2](http://www.ncbi.nlm.nih.gov/sites/entrez?cmd=search&db=nucleotide&dopt=GenBank&term=NM_001079911.2) | 2645 | tcagcatcctgctcatgttc | 112 | 106 |  |
| hDMP1-b218 |  |  | agccaaatgacccttccatt | 218 |  |  |
| DMP1 hF | [NM_001079911.2](http://www.ncbi.nlm.nih.gov/sites/entrez?cmd=search&db=nucleotide&dopt=GenBank&term=NM_001079911.2) | 2645 | tgggcatagatttcctctttg | around 70 | 129 | 6087 |
| DMP1 hR |  |  | aaggaacatgagcaggatgc |  |  |  |

Table S4. List of primer for qRT-PCR of human gene.

|  | sequence, 5' to 3' | note |
| --- | --- | --- |
| Snourd43-hmr | cttattgacgggcggacagaaac | GC:12/23, Tm:58 |
| Snord48-h/m | GAGTGTGTCGCTGATGCCA | GC:11/19, Tm:57.8 |
| Snord48-h | TCACCGCAGCGCTCTGACC | GC:13/19 |
| sonrd66-m/h | ccacgtgtctgggccactga | GC:13/20 |
| snord66-mmu | tctgtcagtgccacgtgtct | GC11/20 |
| RNU6(SBI)-h | cgcaaggatgacacgcaaattc | GC:11/22, Tm:57.3 |
| miR-16-1 h/m | TAG CAG CAC GTAAATATTGGCG |  |
| mir-30a h/m | TgTaaacaTccTcgacTggaag |  |
| mir-30b m/h | TGTAAACATCCTACACTCAGCT |  |
| mir-30c-1/2 mmu | TgTaaacaTccTacacTcTcagc |  |
| mir-30d h/m | TGTAAACATCCCCGACTGGAAG |  |
| mir-30e m/h | TGTAAACATCCTTGACTGGAAG |  |
| miR-155 m | TTAATGCTAATGTGATAGGGGT | Tm:51 |
| miR-322/424 | CAGCAGCAATTCATGTTTTGGA | Tm:54 |
| miR-503 | TAGCAGCGGGAACAGTACTGCAG | Tm:61 |
| miR-541 | aaaggattctgctgtcggtcccact |  |

Table S5. List of primers for qRT-PCR of miRNA.
